# Detection of Differentially Expressed Cleavage Site Intervals Within 3′ Untranslated Regions Using CSI-UTR Reveals Regulated Interaction Motifs

**DOI:** 10.3389/fgene.2019.00182

**Published:** 2019-03-12

**Authors:** Benjamin J. Harrison, Juw Won Park, Cynthia Gomes, Jeffrey C. Petruska, Matthew R. Sapio, Michael J. Iadarola, Julia H. Chariker, Eric C. Rouchka

**Affiliations:** ^1^Department of Biomedical Sciences, Center for Excellence in the Neurosciences, College of Osteopathic Medicine, University of New England, Biddeford, ME, United States; ^2^Department of Anatomical Sciences and Neurobiology, University of Louisville, Louisville, KY, United States; ^3^Kentucky Biomedical Research Infrastructure Network Bioinformatics Core, Louisville, KY, United States; ^4^Department of Computer Engineering and Computer Science, Speed School of Engineering, University of Louisville, Louisville, KY, United States; ^5^Kentucky Spinal Cord Injury Research Center, University of Louisville, Louisville, KY, United States; ^6^Department of Neurological Surgery, University of Louisville, Louisville, KY, United States; ^7^Department of Perioperative Medicine, Clinical Center, National Institutes of Health, Bethesda, MD, United States

**Keywords:** alternative polyadenylation, polyadenylation, polyA, RNA-Seq, polyA, UTR, 3′UTR

## Abstract

The length of untranslated regions at the 3′ end of transcripts (3′UTRs) is regulated by alternate polyadenylation (APA). 3′UTRs contain regions that harbor binding motifs for regulatory molecules. However, the mechanisms that coordinate the 3′UTR length of specific groups of transcripts are not well-understood. We therefore developed a method, CSI-UTR, that models 3′UTR structure as tandem segments between functional alternative-polyadenylation sites (termed cleavage site intervals—CSIs). This approach facilitated (1) profiling of 3′UTR isoform expression changes and (2) statistical enrichment of putative regulatory motifs. CSI-UTR analysis is UTR-annotation independent and can interrogate legacy data generated from standard RNA-Seq libraries. CSI-UTR identified a set of CSIs in human and rodent transcriptomes. Analysis of RNA-Seq datasets from neural tissue identified differential expression events within 3′UTRs not detected by standard gene-based differential expression analyses. Further, in many instances 3′UTR and CDS from the same gene were regulated differently. This modulation of motifs for RNA-interacting molecules with potential condition-dependent and tissue-specific RNA binding partners near the polyA signal and CSI junction may play a mechanistic role in the specificity of alternative polyadenylation.

Source code, CSI BED files and example datasets are available at: https://github.com/UofLBioinformatics/CSI-UTR

## Introduction

Detecting differential expression of regions of the 5′ and 3′ untranslated regions (UTRs) is of great importance for understanding the processes of transcription, translation, and transcript localization. Specifically, shortening and lengthening of 3′ UTRs through alternative polyadenylation (APA) on a global and gene-specific scale has been associated with cell proliferation, cancer, development, and cell differentiation (Di Giammartino et al., 2011). Approaches for detecting and characterizing alternative splicing events in the UTRs provide the opportunity to increase the utility, impact, and efficiency of NGS transcriptomic experiments. Importantly, the expansion of the known repertoire of UTRs improves the accuracy of alignment which is critically important for quantification of gene products using RNA-Seq. Beyond this, the study of these UTR splicing events represents an understudied but rich landscape for potential transcriptional regulation with broad implications for dynamic biological processes in many fields of research.

More recently, approaches to measure alternative splicing in coding sequence (CDS) regions have appeared, including methods that analyze differential expression at the exon level (Katz et al., 2010; Wu et al., 2011; Shen et al., 2012, 2014; Hu et al., 2013; Hartley and Mullikin, 2016). The decreasing costs in sequencing, along with development of APA sequencing methods (polyA-Seq) (Fox-Walsh et al., 2011; Shepard et al., 2011; Derti et al., 2012) now allow for a more thorough understanding of the complete transcript in (potentially) all its forms, including the 5′ and 3′ UTRs which play significant roles in both transcriptional and translational regulation.

One of the limits of differential expression approaches is the reliance on gene and transcript annotations (Consortium, 2014). When considering a well-studied species, the CDS regions are likely to be fairly well-annotated. However, the UTRs are generally poorly constructed, even when the CDS regions are well-described. As a case in point, sequencing technologies were used to understand 3′ UTRs in *C. elegans* (Mangone et al., 2010). Prior to their work, less than half of the genes in WormBase (Howe et al., 2016) had annotated 3′ UTRs, while with a relatively low coverage they were able to construct 3′ UTRs for 73% of all genes, including over 7,000 previously unannotated APA sites. Other studies have proceeded to capture APA within tumor types (Xia et al., 2014) and within mammalian cell lineages (Wang et al., 2013).

**Figure 1:**
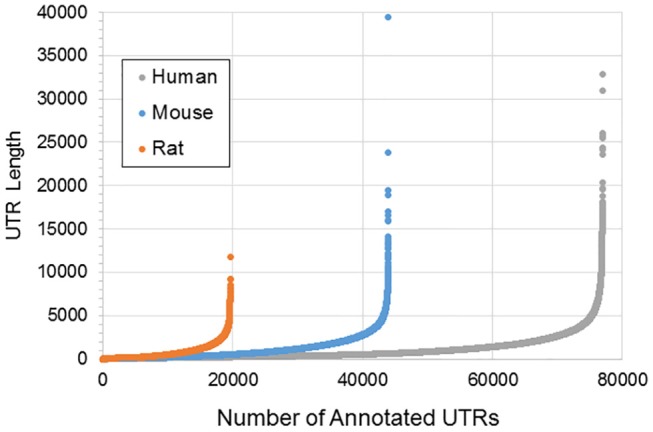
Length and distribution of UTRs for human, mouse, and rat. Shown in the x-axis is the cumulative number of total UTRs. The y-axis for each point represents the longest annotated length from the stop codon for each Ensembl gene, with genes sorted from shortest to longest along the x-axis.

Figure 1 illustrates the poor annotation and inconsistency in 3′UTR structure available in databases, showing that the number of annotated 3′ UTRs for rat is about 25% that for human, and the number for mouse is a little more than 50% the number for human. It also illustrates the length of such 3′UTRs, which can be as long as ~10,000–40,000 bp. A summary of the annotated UTRs is given in Table 1.

**Table 1:** Summary statistics of annotated 3′ UTRs in the human, mouse, and rat.

**Species**	**Annotated 3′ UTRs^a^**	**Median length**	**Maximum length**	**Detected APAs^b^**
Human	76,946	492	32,870	439,390
Mouse	43,997	615	39,397	127,014
Rat	19,620	479	11,772	200,593

a *Does not include APA sites—only the longest 3′ UTR is represented for each transcript*.

b *As detected by Derti et al. (2012), including novel APA sites*.

Derti et al. (2012) developed the polyA-Seq approach that was applied to five mammalian genomes in order to help address this issue. The number of detected rat APAs using polyA-Seq is much larger than mouse (Table 1), which is counter to the number of annotated 3′ UTRs, further highlighting the limitation of canonical annotations.

The choice of the site for adding a polyA tail onto an mRNA transcript is largely driven by a complex of proteins, including the cleavage/polyadenylation specificity factor (*CPSF*) which binds to a motif typically 10–30 nucleotides upstream of the cleavage site (Bienroth et al., 1993). The canonical binding sequence for *CPSF* is the hexamer AAUAAA, but alternative hexamer binding sequences are functional as well. The top 10 hexamers have recently been shown to account for 98% of all polyA sites, and their usage is highly tissue dependent, an observation found across multiple mammalian species (Derti et al., 2012).

Recent advances in understanding translational control mechanisms such as miRNA (Carrington and Ambros, 2003), AU rich elements (AREs) (Shaw and Kamen, 1986; Chen and Shyu, 1995), cytoplasmic polyadenylation elements (CPEs) (Mcgrew et al., 1989), and localization binding elements (Jansen, 2001) illustrate the important role that the 3′ UTR plays, particularly in processes such as development, embryonic axis formation, neurogenesis, and erythropoiesis where post-transcriptional control is critical in controlling mRNA stability, localization, and translation (Kuersten and Goodwin, 2003). A disproportionate number of UTRs showing condition and/or location specific differential expression have been found within the nervous system (Mercer et al., 2011, 2012). Studies have shown that well over 50% of all mammalian genes have multiple polyadenylation sites, indicating alternative splicing in the 3′UTR that may or may not be associated with changes in the coding regions (Tian et al., 2005). Therefore, it is highly likely that changes in the structure of the 3′ UTR of an mRNA will greatly affect the expression or sub-cellular localization of a particular transcript, even in cases where the coding region remains the same.

For instance, brain-derived neurotrophic factor (*BDNF*) has two alternatively polyadenylated transcripts in the brain, differentiated by short and long 3′ UTRs. The role of the 3′ UTR appears to be localization, with the short 3′ UTR mRNAs restricted to somata, and long 3′ UTR mRNAs localized in dendrites (An et al., 2008). Additional genes including *ARC* (Kobayashi et al., 2005), *MAP2* (Blichenberg et al., 1999), α*CAMKII* (Mori et al., 2000), *SHANK1* (Böckers et al., 2004), and vasopressin (*AVP*) (Prakash et al., 1997) contain dendrite targeting elements (DTEs) in their 3′ UTRs as well, which has been experimentally demonstrated as a prerequisite for dendrite localization via an association with the protein *CBF-A* (Raju et al., 2011). Sensorin contains a 66 nucleotide (nt) 3′ UTR localization element (LE) that is sufficient for localization to distal neurites (Meer et al., 2012). Cytochrome C oxidase IV (*COXIV*) contains a signal in its 3′ UTR that serves as a necessary and sufficient condition for transport to distal axons (Aschrafi et al., 2010). A 60 nt segment of amphoterin (*HMGB1*) mRNA is sufficient for its localization in axons of cultured sensory neurons (Merianda et al., 2015). The 3′ UTR of β-actin is sufficient to target mRNA for axonal transport (Willis et al., 2011) based on a conserved zip code element (Kislauskis et al., 1994). An additional study has shown the association of a number of genes with RNA binding proteins, including the zip code binding protein, *ZBP1* (Patel et al., 2012). Other studies have suggested the potential role of G-quadraplexes located in the 3′ UTR of mRNAs localized to neurites (Subramanian et al., 2011). All of these examples underscore the functional importance of regions within the 3′ UTR.

Both lengthening and shortening of the 3′ UTRs are important processes during development, regulating the number of sites available for interactions with RNA binding proteins. In Drosophila, a subset of neural specific genes exhibit elongation of their 3′ UTRs during embryogenesis, producing 3′ UTRs that are 20-fold longer than typical mRNAs (Hilgers et al., 2011). A similar study in mouse (Ji et al., 2009) showed that mRNAs expressed in the mouse brain during embryonic and postnatal development tend to have longer 3′ UTRs than other tissues. In addition, this study showed an 8- to 20-fold increase in the number of genes with lengthened 3′ UTRs during differentiation of C2C12 myoblast cells to myotubes. Shortened 3′ UTRs also play a role in translational control. Proliferating cells express mRNAs with shortened 3′ UTRs (Sandberg et al., 2008), allowing them to have fewer miRNA target sites which protects them against degradation by dicer. It has been shown that shortened mRNAs activate oncogenes, have an increased stability, and are transcribed 2.6 times more efficiently (Mayr and Bartel, 2009). Tumors expressing shorter 3′ UTRs have been shown to be more aggressive in nature, and gene expression signatures based solely on 3′ UTRs are strong predictors of survival (Lembo et al., 2012). The insertion of a transposon within the 3′ UTR of the *COMT* gene in certain strains of mice has been demonstrated to induce a shortened 3′ UTR isoform associated with increased protein expression in the prefrontal cortex and hippocampus (Li et al., 2010). In addition to the 3′ UTR serving as a *cis* mechanism for regulating translation of an mRNA sequence, a recent study has suggested that post-translational processing of the 3′ UTR can also produce non-coding RNAs termed uaRNAs (3′ UTR-associated RNAs) that can act in *trans* to regulate gene expression (Flynn et al., 2011; Mercer et al., 2011).

Recent studies have considered the roles that APA has within breast cancer. These studies indicate that 3′ UTR signatures can be used to define a highly metastatic subgroup of triple-negative breast cancer (Wang et al., 2016). This is hypothesized to occur due to an upregulation of *CSTF2* in response to *EGF*, resulting in shortening of 3′UTRs (Akman et al., 2015). Examination of two breast cancer cell lines shows a complex regulation of APA, with MCF7 transcripts showing broad 3′ UTR truncation, and MB231 exhibiting elongated 3′UTRs (Fu et al., 2011).

## Materials and Methods

### Sequencing Ends of Transcripts

Recently developed methods for sequencing the ends of mRNA use a poly-dT primer to detect the polyA tail, with sequencing extending into the CDS for gene identification. The resulting sequences thus produce 3′-biased cDNA libraries which can be further explored for alternative polyadenylation site detection. In the case of PAS-seq (Shepard et al., 2011) and MAPS (Fox-Walsh et al., 2011), the universal primer used is of the form T_20_VN, representing 20 consecutive T's (complementary to the polyA tail), followed by a non-T nucleotide, and ending with any nucleotide. This pattern allows for the precise determination of the location of the beginning of the polyA tail, thus indicating the APA site. For PolyA-Seq, the primer is modified to T_10_VN, allowing for shorter polyA tails and more favorable hybridization kinetics (Derti et al., 2012). More recent approaches have considered the use of these data for modeling polyadenylation sites (Ji et al., 2015; Szkop and Nobeli, 2017).

### Computational Approaches to Detecting Differential UTR Expression

The current state of analysis of differential 3′ UTR expression is nascent. Methods for analysis of 3′ UTRs have been focused mainly on detecting the extent of the 3′ UTR landscape in order to improve annotation for transcript assembly, including IsoSCM (Shenker et al., 2015) which employs change-point models for detecting differences in RNA-Seq coverage (Zhang and Wei, 2016); KLEAT (Birol et al., 2015) which uses poly(A) tails represented in RNA-Seq data to define the ends of transcripts, and GETUTR, which defines 3′ UTR boundaries using heuristic and regression approaches (Kim M. et al., 2015). Other methods look at APA site switching from long to short forms using statistical methods such as an independent test and linear trend test (Li et al., 2015), hidden Markov models (Lu and Bushel, 2013), change-points (Wang et al., 2014), or consider the presence or absence of alternative tandem APAs, as with 3USS (Le Pera et al., 2015). Very recent approaches have been taken to catalog polyadenylation sites using RNA-Seq data (Yeh et al., 2017; Arefeen et al., 2018; Ha et al., 2018; Ye et al., 2018). To the best of our knowledge, none of the approaches provide statistical methodologies for considering differential expression of regions of 3′UTRs associated with alternative polyadenylation outside of determining gross shortening or lengthening events. In order to elucidate mechanisms, much greater resolution is needed. We therefore have developed an approach, CSI-UTR, which builds cleavage-site intervals (CSIs) based on polyA-Seq datasets for defining observable APA sites. This information is then used to determine significant changes in CSIs within 3′ UTRs for RNA-Seq datasets.

### CSI-UTR

**Figure 2:**
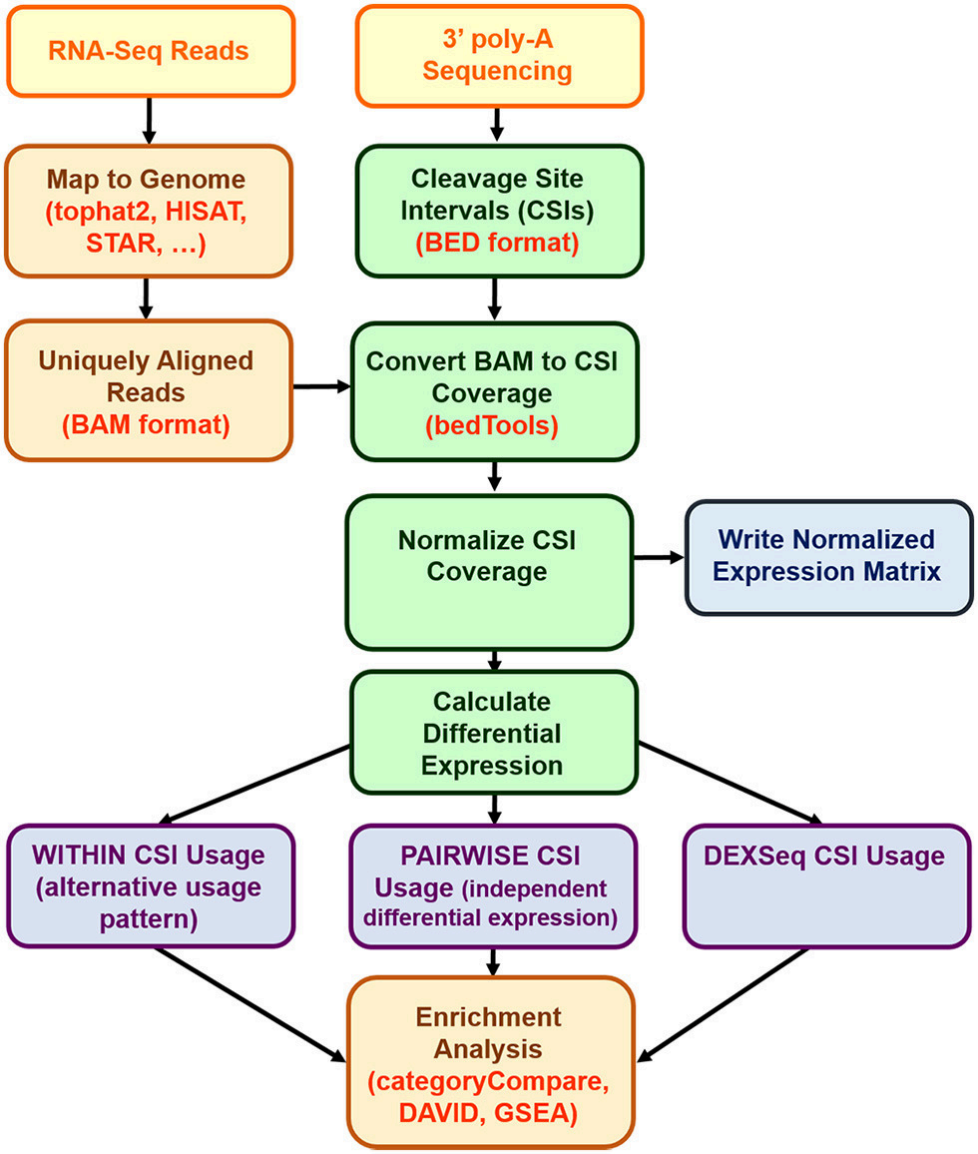
CSI-UTR process diagram. (Yellow = external data sources; orange = external programs; green = CSI-UTR algorithms; purple = differential expression results; blue = output files). The input into CSI-UTR requires two sets of inputs: uniquely aligned input reads in BAM format which can be determined from raw RNA-Seq reads mapped to the corresponding reference genome; and a set of CSIs defined in BED file format. The CSIs are pre-computed for the hg38, mm10, and rn6 reference genomes using publicly available polyA-Seq data. The aligned reads are then mapped to CSIs and are normalized, resulting in a normalized expression matrix that is used to calculate differential expression using the CSI-UTR WITHIN, PAIRWISE, and DEXSeq methods. Once differentially expressed CSIs are identified, their associated genes can be used for further downstream enrichment analysis.

Our approach, CSI-UTR, requires two sets of inputs, (1) the CSIs to be examined in BED format, and (2) the sequence reads. An overview of the approach of CSI-UTR is given in Figure 2. We first define sets of CSIs as detailed in the section “Defining Cleavage Site Intervals (CSIs)”. Once these CSIs are set for a given species, analysis can proceed on any given dataset as detailed in the section “Computational approaches to detecting differential UTR expression”.

#### Defining Cleavage Site Intervals (CSIs)

**Figure 3:**
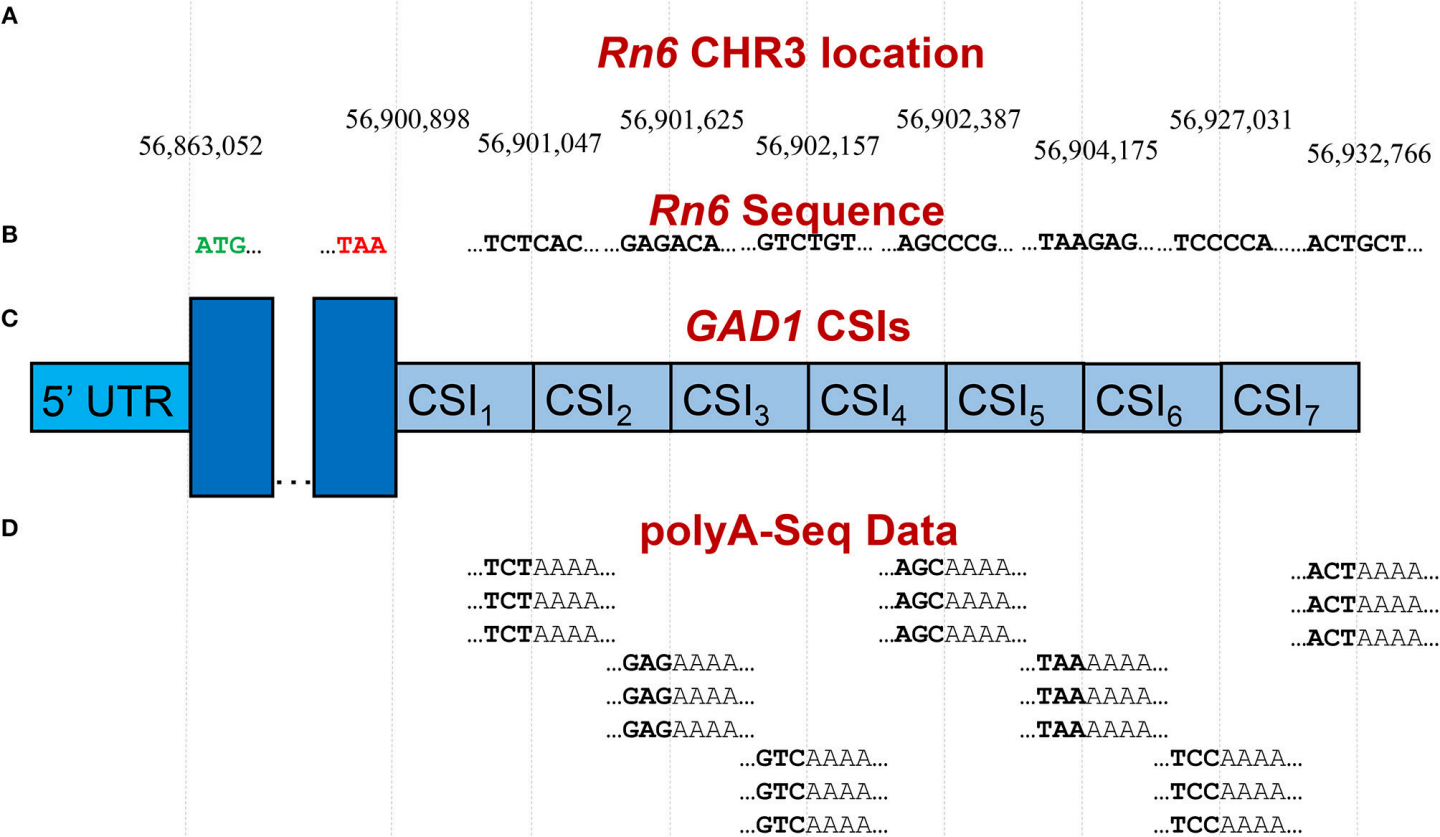
Example of CSI structure for the *GAD1* gene based on the rat Rn6 assembly. **(A)** Key chromosomal locations on Rn6 chromosome 3, including cleavage site locations (indicating CSIs) (not to scale). **(B)** Rn6 reference genome sequence at boundary locations. **(C)** Structure of the seven CSIs identified in the 3′ UTR region of *GAD1* by CSI-UTR, based on polyA-Seq data. **(D)** Example polyA-Seq reads showing polyA sequences at cleavage sites. Based on the evidence of cleavage sites provided by the polyA-Seq data in **(D)**, CSI-UTR identifies seven cleavage site intervals (CSIs), beginning immediately after the distal most stop codon at position CHR3:56,90,898. CSI-UTR extends potential GAD1 transcripts to CHR3:56,932,766, well past the annotation provided by RefSeq entry NM_017007.1 which extends to CHR3:56,902,139.

Publicly available RNA-Seq datasets, along with 3′ polyA-Seq data, allows for a more accurate detection of the true 3′ ends of transcripts. We define a cleavage site interval (CSI) for a particular 3′ UTR as a region in-between two functional alternative polyadenylation sites. Using polyA-Seq reads from the study by Derti et al. (2012) (GEO series GSE30198; SRA accession SRP007359) which performed sequencing on a variety of tissues from human, mouse, and rat, we developed a pipeline for defining CSIs in these species. An overview of the CSI structure within the 3′ UTR of the rat ***GAD1*** gene is provided in Figure 3.

#### Preparing Regions of Interest Using Gene Transfer Files (GTFs)

The first step in determining cleavage site intervals for a particular species is to prepare potential intervals where alternative polyadenylation can occur for each known protein coding gene. This process begins with obtaining an appropriate GTF for the organism build of interest. In our case, we downloaded GTF files for human (hg38), mouse (mm10), and rat (rn6) from Ensembl's ftp site (usaeast.ensembl.org/info/data/ftp/index.html).

For human, Ensembl release 82 was used, while Ensembl release 84 was used for mouse and rat. Due to the incomplete annotations for the rat transcriptome, we created a second set of GTFs for the rat which additionally incorporated RefSeq annotations. Stop codons and exons were parsed from the GTF files into separate stop codon and exon files for each organism, separated by the exon's strandedness (coding or template strand). All exons annotated as non-coding (such as microRNAs, lncRNAs, pseudogenes, 5′ and 3′ UTRs) were parsed into a non-coding exon GTF for both the coding and template strands, and stored separately. A BED file (Quinlan, 2014) was created for each resulting GTF. The BED files were searched, and overlapping exons were concatenated and stored into a new BED file. All exons were searched against the stop codons previously parsed to determine terminal exons (exons containing a stop codon) in both the coding and template strands. The stop codons were parsed to determine overlapping stop exons in a pairwise fashion for both the coding and template strands. Overlapping stop exons were merged based on name and positional overlap and the most distal stop codon location was determined. The region between the distal stop codon and the beginning (or end, if occurring on opposite strands) of the next gene was determined based on current annotations. The resulting regions, which included current annotated 3′ UTRs as well as intergenic locations, were stored as intervals and marked for potential overlap with polyA-Seq data. Such an approach allowed for the extension of known 3′ UTRs given the resulting polyA-Seq data.

#### Determining PolyA Ends of Protein-Coding mRNAs

The 3′ ends of protein-coding mRNAs was determined using polyA-Seq data generated from a previous study (Derti et al., 2012) for human, mouse, and rat. This approach can be more generally applied to any organism of interest where an appropriate depth of 3′ sequencing data is available. While this approach is limited to those 3′ UTRs that have already been discovered, performing 3′ sequencing on the same tissue of interest in the experimental manipulation could be performed to ensure complete annotation of novel UTR splicing. Sequence data was downloaded from the Sequence Read Archive ftp site, and converted to fastq format using the fastq-dump tool from the SRA toolkit (Leinonen et al., 2011). These datasets (listed in Table 2) were analyzed separately in order to allow for tissue specificity and were later concatenated to generate a broader database of known UTRs. The resulting fastq files were trimmed for sequence quality using Trimmomatic v0.33 (Bolger et al., 2014) with the parameters “ILLUMINACLIP:TruSeq2-SE.fa:2:30:10 LEADING:20 TRAILING:20 SLIDING WINDOW:3:30 MINLEN:25.”

**Table 2:** SRA polyA-Seq data utilized from Derti et al. (2012).

**Organism**	**SRA Identifier**	**Tissue**
Hs	SRR299106	Brain
Hs	SRR299107	Kidney
Hs	SRR299108	Liver
Hs	SRR299109	MAQC Brain1
Hs	SRR299110	MAQC Brain2
Hs	SRR299111	MAQC Universal Human Reference (UHR) 1
Hs	SRR299112	MAQC UHR2
Hs	SRR299113	Muscle
Hs	SRR299114	noVN (UHR)
Hs	SRR299115	Testis
Hs	SRR299116	UHR
Mm	SRR299117	Brain
Mm	SRR299118	Kidney
Mm	SRR299119	Liver
Mm	SRR299120	Muscle
Mm	SRR299121	Testis
Rn	SRR299122	Brain
Rn	SRR299123	Testis

Genomes for mouse, human, and rat were downloaded from the UCSC genome resource (Kent et al., 2002). Genome versions used include hg38 (human), mm10 (mouse), and rn6 (rat). Each of the SRA fastq files were mapped to the corresponding genome using tophat v2 (Kim et al., 2013) with the Ensembl genome GTF as a guide for known exon junctions. Up to two hits per read (parameter “-g = 2”) were used in order to decrease the likelihood of false alternative polyadenylation sites. In addition, the parameter settings “-p4 —library-type fr-firststrand —no-coverage-search” were used. After the sequences were mapped, the bam alignment files were converted to SAM files using samtools view (Li et al., 2009). These SAM files were then parsed and split into template and coding strand alignments, filtering out reads that did map to unique positions on the genome. The SAM files were subsequently converted to BED alignment files containing information concerning the chromosome, chromosome start, chromosome end, read name, score, and strand. Peak polyA locations were constructed based on read beginning positions from the BED file. The number of reads starting at each chromosomal location were tallied. A region surrounding the polyA peaks was constructed consisting of 40nt upstream and 30nt downstream of each peak location, based on suggested settings for the R cleanUpdTSeq package (Sheppard et al., 2013). Sequences for these regions were obtained using bedtools getfasta (Quinlan, 2014). The resulting peak results were used as input into cleanUpdTSeq to determine which peaks were likely due to true polyA events from mRNA sequences, and not from internal priming events as a result of homopolymer runs within the CDS. The scores from cleanUpdTSeq were added to the BED files for each region identified. The polyA sites having a positive score (likely true mRNA polyA events) were clustered together if they were within 30 bp of each other. As shown in Supplemental Figure 1, over 90% of clustered polyA sites occur within 20 kb of the distal-most stop codon for hg38, mm10, and rn6. For the human and mouse genomes, <5% of all clustered polyA sites extend past 40 kb.

#### Assigning PolyA Sites to Gene Regions

Clustered polyA sites were searched against the terminal exon intervals to assign each polyA cluster to its closest gene based on the region between the distal stop codon/terminal exon and the next known coding exon. Cleavage site intervals for each gene were then constructed with the first location corresponding to the distal stop codon, and the last location marked by the distal polyA cluster occurring within the interval. Each CSI corresponds to a region between polyA clusters (or the distal stop codon) for a particular gene. The CSI annotations for each organism were then constructed and stored as BED files (Zhang, 2016) which are tab-delimited files. Table 3 shows an example for the CSIs for the *GAD1* gene in modified BED format in the rat rn6 assembly. In the series of CSIs detected, the longest UTR in the *GAD1* gene was 1719 nt. In addition to the traditional BED fields, the name field is modified to contain the CSI identifier in the format GeneName:StopCodonPosition_CSIBeginLocation-CSIEndLocation; and an additional field denotes the gene identifier, typically from Ensembl (Yates et al., 2016) or RefSeq (O'leary et al., 2016).

**Table 3:** Example CSI BED file for *GAD1*.

**Chr**	**Chr Begin**	**Chr End**	**CSI identifier**	**1**	**Strand**	**Gene**	**Symbol**
Chr3	56900898	56901047	ENSRNOG00000000007:56900898_56900898-56901047	1	+	ENSRNOG00000000007	*Gad1*
Chr3	56901047	56901625	ENSRNOG00000000007:56900898_56901047-56901625	1	+	ENSRNOG00000000007	*Gad1*
Chr3	56901625	56902157	ENSRNOG00000000007:56900898_56901625-56902157	1	+	ENSRNOG00000000007	*Gad1*
Chr3	56902157	56902387	ENSRNOG00000000007:56900898_56902157-56902387	1	+	ENSRNOG00000000007	*Gad1*
Chr3	56902387	56904175	ENSRNOG00000000007:56900898_56902387-56904175	1	+	ENSRNOG00000000007	*Gad1*
Chr3	56904175	56927031	ENSRNOG00000000007:56900898_56904175-56927031	1	+	ENSRNOG00000000007	*Gad1*
Chr3	56927031	56932766	ENSRNOG00000000007:56900898_56927031-56932766	1	+	ENSRNOG00000000007	*Gad1*

*The CSI Identifier field (fourth column) is in the format GeneName:StopCodonPosition_CSIBeginLocation-CSIEndLocation*.

### Detecting Differential Expression

CSI-UTR takes as its inputs two sets of data: (1) a file containing a list of cleavage site intervals defined in BED format (Table 3) and (2) a set of files, one for each sample, containing reads aligning uniquely to the genome of interest in BAM format. The BED file is constructed once per genome, as outlined in section “Sequencing ends of transcripts”. Pre-computed BED files are also available for download for human, rat, and mouse at https://github.com/UofLBioinformatics/CSI-UTR. BAM files can be constructed for a particular RNA-Seq experimental setup using a splicing-aware mapping tool of interest, such as STAR (Dobin et al., 2013), HISAT (Kim D. et al., 2015) MapSplice (Wang et al., 2010) or tophat2 (Kim et al., 2013). In order to ensure high confidence that the reads belong to a particular CSI we filtered for only uniquely-mapping reads. Using tophat2, the command was:

tophat2 –no-coverage-search –g=2 \

<bowtie_index> <fastq_reads>

Using the BED and BAM files as input, the alignment file is converted into a CSIcoverage file using the CSI BED intervals and the coverageBed utility from BEDTools (Quinlan and Hall, 2010; Quinlan, 2014). The resulting raw CSI counts are normalized to a counts per million (CPM) value for each sample, resulting in a comparable score for each CSI. These normalized values are written as a normalized CSI expression matrix, and are marked for further differential expression analysis.

Significant usage of each CSI between two different experimental conditions is calculated using three separate methods: pairwise CSI usage (PAIRWISE), within CSI usage (WITHIN), and DEXSeq determined differential CSI expression (DEXSeq). The following variables for CSI counts are calculated, where *CPM* is the normalized Counts Per Million value. In this case, 0.5 is added as a pseudocount to adjust for zero values:

(1) $$\begin{array}{r} a_{ij}=\left\lfloor CPM+0.5\right\rfloor \;for\;CSI_{j}\text{ in condition }1,\text{ replicate }i \end{array}$$

(2) $$\begin{array}{r} b_{ij}=\left\lfloor CPM+0.5\right\rfloor \;for\;CSI_{j}\text{ in condition }2,\text{ replicate }i \end{array}$$

(3) $$\begin{array}{r} A_{j}=\sum_{i=1}^{numRep}a_{ij};B_{j}=\sum_{i=1}^{numRep}b_{ij} \end{array}$$

#### Pairwise CSI Differential Expression (PAIRWISE)

In the pairwise significance test, significance is computed between the two conditions, A and B, for a specific CSI, *CSI*_*i*_, using a student's *t*-test with input values set as two vectors, *V*_1*i*_ and *V*_2*i*_ representing the indivudal replicate CPM values as follows:

(4) $$\begin{array}{r} V_{1i}=\left(a_{1i},\;a_{2i},\;\ldots ,\;a_{mi}\right) \end{array}$$

(5) $$\begin{array}{r} V_{2i}=\left(b_{1i},\;b_{2i},\;\ldots ,\;b_{ni}\right) \end{array}$$

Where *m* is the number of replicates in conditon 1, and *n* is the number of replicates in condition 2. This approach will determine if *CSI*_*i*_ is differentially expressed between conditions. However, this is done independently of the expression of the other CSIs and of the CDS itself. Therefore, if *CSI*_*i*_, is differentially expressed using this methodology, it may be indicative of a global change in the expression of the gene itself and does not necessarily indicate APA usage. However, the difference in gene level expression should be recognizable from the transcript quantification itself. Given this limitation, we further developed two within condition (and within gene) approaches that consider the overall usage rates of a particular CSI within a condition relative to the rate of utilization of other CSIs within the same gene and compare these rates across conditions. These additional approaches thus separate out the overall gene expression which is not considered as a factor.

#### Within CSI Usage Differential Expression (WITHIN)

Since the overall goal is to determine which CSIs are differentially expressed, indicating APA usage, a more robust measure of significance was adopted from the MATS and rMATS approaches for determining exon skipping events (Shen et al., 2012, 2014). In short, this approach compares the overall percentage of reads within a UTR that are incorproated within a specific CSI. The percentage of reads belonging to that CSI in condition 1 is compared to the percentage of reads belonging to that CSI in condition 2 using a Fisher exact test. The statistics for the within CSI usage model are calculated as follows: for each *CSI*_*i*_, a usage, *ψ* is calculated for condition *A* and *B* using the following equations:

(6) $$\begin{array}{r} \psi_{A_{i}}=\frac{A_{i}}{\sum_{j=1}^{numCSI}A_{j}};\;\psi_{B_{i}}=\frac{B_{i}}{\sum_{j=1}^{numCSI}B_{j}} \end{array}$$

A difference in usage, Δ*ψ*, is then calculated as:

(7) $$\begin{array}{r} \Delta \psi_{i}=\psi_{A_{i}}-\psi_{B_{i}} \end{array}$$

In order to determine the significance for differential usage of each CSI region, a *p*-value is calculated for each CSI using a Fisher exact test with a contingency table as follows:

(8) $$\begin{array}{c} {\text{Pval}}_{\text{CSI}}\text{= Fisher Exact Test}\\ (A_{i},\left(\sum_{j=1}^{numCSI}A_{j}\right)-A_{i},\\ B_{i},\left(\sum_{j=1}^{numCSI}B_{j}\right)-B_{i}) \end{array}$$

**Figure 4:**
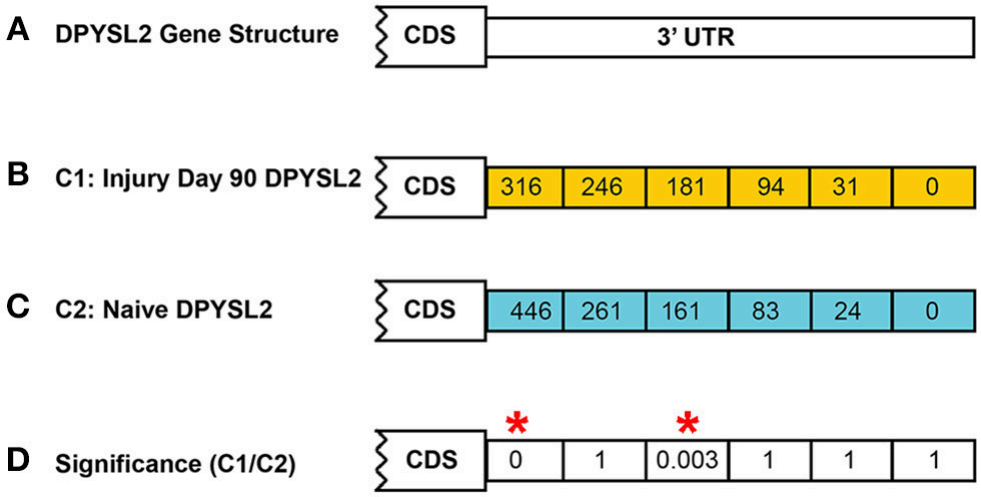
Example of CSI significance calculation. Shown is the calculation of the FDR-corrected significance for dihydropyrimidinase like 2 (*DPYSL2*) in the rat injury day 90 vs. naïve. A total of six possible CSIs have been identified for *DPYSL2* **(A)**. Normalized CSI counts for the injury and naïve conditions are shown in **(B,C)**, respectively. FDR-corrected significance values for each CSI are shown in **(D)**. Statistically significant regions, as determined by Fisher's exact test, are highlighted by a red*.

In both models of differential expression, each *p*-value is corrected for false discovery rate (FDR) using the Benjamini–Hochberg correction (Hochberg and Benjamini, 1990). An example calculation is shown in Figure 4 for the *DPYSL2* gene which has six CSIs. In this case, the significance value shown is the FDR-corrected value.

#### DEXSeq Formatted Results (DEXSeq)

While the WITHIN method of differential expression utilizing a Fisher exact test as described in the previous section is a similar approach taken by previous methods for detecting alternative splicing events (Shen et al., 2012) and 3′ UTR lengthening and shortening events (Xia et al., 2014), it has the limitation of removing replicate data, and therefore reducing the effect of variance and dispersion on significance detection. In order to incorporate replicate information, the approach taken by DaPars (Xia et al., 2014) involves pairwise comparisons between each replicate in condition 1 to each replicate in condition 2. However, this still ignores the overall variance for each region, thus giving too much weight to outliers. If the samples can be paired (such as paired tumor-healthy datasets), then a modified Cochran-Mantel-Haenszel test could be used. However, this requires a specific set of conditions where the number of samples in both conditions is the same. A more comprehensive alternative approach involves an estimate of dispersion across all samples. DESeq2 (Love et al., 2014) uses a generalized linear model for detecting differential expression in this fashion. Building off of this notion, DEXSeq (Anders et al., 2012) was constructed to determine differential exon usage for cassette exons which assumes differential splicing using only inclusion/exclusion events without considering alternative 5′ and 3′ splice sites. Since CSIs can be thought of as cassette exons in the 3′ UTR, once the CSIs have been constructed and their counts determined for each sample, their differential expression can be computed using the DEXSeq algorithm. Thus, we provide a third approach which uses a modified DEXSeq pipeline for the final step in the differential expression process. When the number of replicates and the per sample read number is small, the WITHIN methodology tends to produce more significantly-different CSIs than the DEXSeq approach. However, as these numbers (and thus the overall power) increase, the number of significant CSIs found by the DEXSeq pipeline increases, with a larger overlap between the WITHIN and DEXSeq methods (results not shown).

### RNA-Binding Motif Enrichment

RNA motif enrichment was performed for significantly differentially expressed CSIs using consensus binding motifs in the ATtRACT database of RNA-binding proteins (Giudice et al., 2016) and motifs with MEME v4.10.0 (Bailey et al., 2015). A 100 bp window surrounding the CSI site was used with significance cutoffs of *p* ≤ 0.05 and FDR ≤ 0.01. Significant CSIs were then shuffled via MEME's fasta-shuffle-letters and used as the background for enrichment analysis. Localized motif enrichment was performed using MEME's centrimo.

## Results

### Genomic CSI Intervals

**Figure 5:**
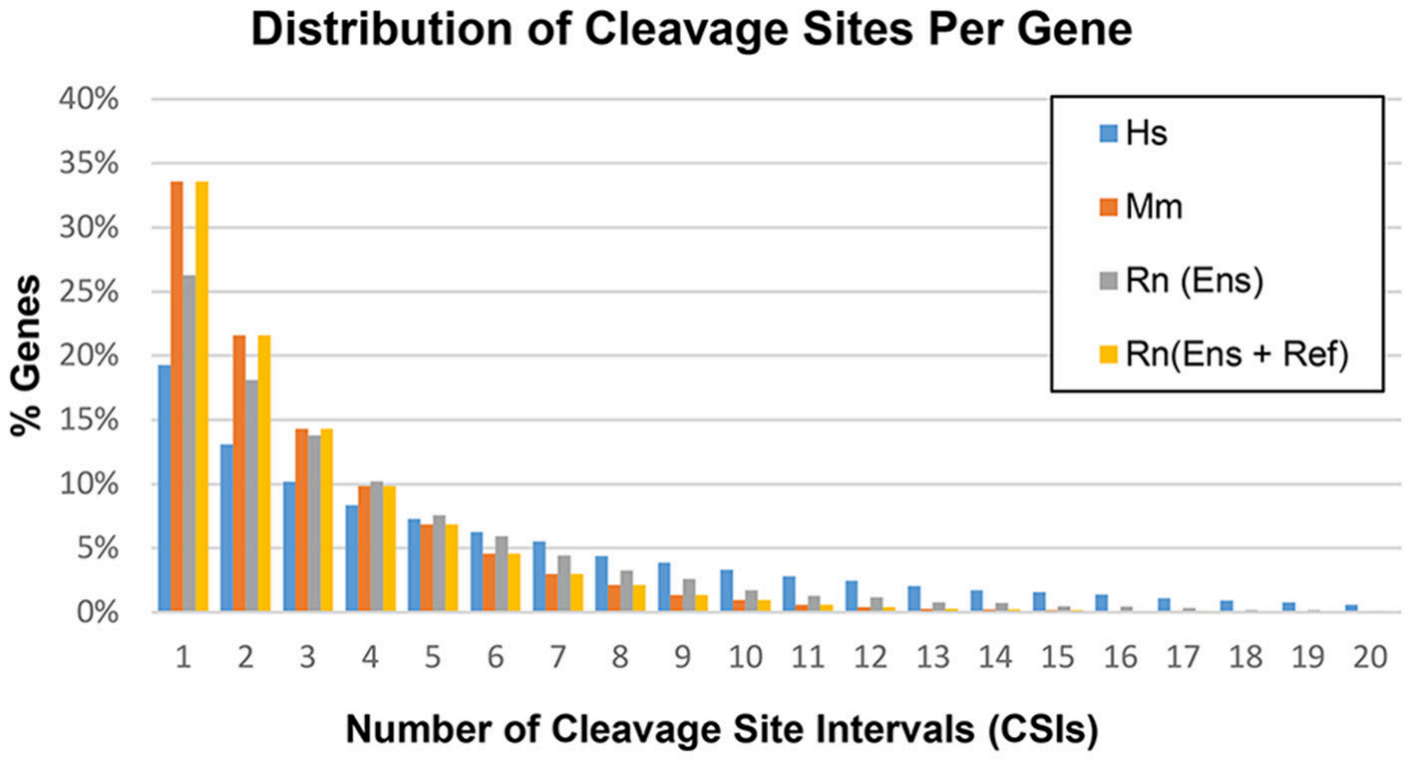
Distribution of cleavage sites per gene in the human (Hs), mouse (Mm), and rat (Rn) transcriptomes.

Based on the methods outlined in the previous section, CSIs were constructed for the following genomes: human hg38, mouse mm10, and rat rn6. The number of CSIs for each genome ranged from 51,489 (Mm) to 106,418 (Hs) (Table 4). Discrepancies in the number of CSIs detected for each organism may reflect true differences, although read depth and tissues studied may play a role as well. The majority of genes had four or fewer CSIs, with the largest fraction having only one or two CSIs (Figure 5).

**Table 4:** Summary of CSIs detected for human, mouse, and rat genome assemblies.

**Org**	**Genes**	**CSIs**	**Genes w/ 4+ CSIs**	**MAX CSIs**
Hs	16,963	106,418	9,749 (57.5%)	77 (*KCTD12*)
Mm	16,819	51,489	5,141 (30.6%)	30 (*CCDC50*)
Rn^a^	16,189	65,764	6,774 (41.8%)	50 (*MAF*)
Rn^b^	18,543	64,706	6,795 (36.6%)	30 (*ATP2B2*)

a *Ensembl gene annotations only*.

b *Ensembl and RefSeq gene annotations*.

However, a number of genes were detected as having a large number of CSIs, with 77 detected for the human potassium channel tetramerization domain containing 12 (*KCTD12*) gene. Not all of these CSIs are expressed in every condition, and are thus filtered internally in our program based on sequence-level evidence of contiguous expression from the terminal end of the CDS (i.e., no zero count CSI gaps are present) when individual experimental conditions are compared. A list of the genes with the highest number of CSIs is provided in Supplemental Table 1.

### Detection of Differentially Expressed CSIs in RNASeq Datasets

In order to test our methodology, we selected three datasets for further analysis (Table 5). The data sets were selected from the nervous system, where 3′ UTR dynamics is known to be robust. All three species for which CSIs were constructed using CSI-UTR are represented. In addition, the human dataset was chosen to be representative of neurological disorders where APA has been shown to be key (de Sauvage et al., 1992; Dickson et al., 2013), while the mouse and rat datasets were chosen because they are commonly-used models for studying repair and response to nerve injury (Yasuda et al., 2014; Guan et al., 2016).

**Table 5:** Datasets analyzed.

**Org**	**SRA**	**Condition**
Hs	SRP056604	Late onset Alzheimer's Disease
Mm	SRP038707	Optic nerve crush
Rn	Unpublished	3 days and 90 days post-injury vs. naïve

For the human late onset Alzheimer's disease (LOAD) samples, 975 genes were shown to have differentially expressed CSIs using our WITHIN approach (FDR < 0.05) (Table 6). Among these are amyloid beta precursor protein (*APP*), which has been previously shown to be alternatively polyadenylated in Alzheimer's patients (de Sauvage et al., 1992). Using the more sensitive DEXSeq approach, 30 genes were determined to have differentially expressed CSIs (FDR < 0.05), including ATP binding cassette subfamily A member 1 (*ABCA1)*, which is a candidate biomarker gene for Alzheimer's disease (Alonso Vilatela et al., 2012; Love et al., 2015).

**Table 6:** Differentially expressed events detected.

**Org**	**Dataset**	**DE-CSIs (genes) WITHIN**	**DE-CSIs (genes) DEXSeq**	**DEGs**
Hs	SRP056604	1622 (975)	32 (30)	105
Mm	SRP038707	339 (245)	78 (68)	338
Rn	3d vs. naïve	9459 (3648)	18487 (6677)	1972
Rn	90d vs. naive	987 (544)	5581 (2866)	672

Among other genes of interest appearing in the set of 30 are aspartoacylase (*ASPA*) which maintains white matter and dysfunction of which is a cause of Canavan disease (Bitto et al., 2007); doublecortin like kinase 1 (*DCLK1*), which is involved in neuron migration and neurogenesis (Deuel et al., 2006); potassium calcium-activated channel subfamily M alpha 1 (*KCNMA1*) which has been associated with LOAD (Grupe et al., 2006) and schizophrenia (Zhang et al., 2006); and synaptophysin like 1 (*SYPL1*) which is involved in neuronal differentiation (Leube, 1994). Gene Ontology Biological Process (GO:BP) enrichment analysis using categoryCompare (Flight et al., 2014) indicates those genes with differentially expressed CSIs are highly enriched for two biological processes (FDR < 0.001), including: substantia nigra development, and cell morphogenesis involved in neuron differentiation (Supplemental Figures 2–4; Supplemental Tables 2,3).

For the mouse CSIs, a dataset from a model of optic nerve crush-induced axonal injury (Yasuda et al., 2014) was considered for analysis. Using the DEXSeq methodology, 68 genes were determined to have differentially expressed CSIs (Table 6) (FDR < 0.05). Among these are alanyl-tRNA synthetase (*AARS*), which is implicated in Charcot-Marie-Tooth disease (Latour et al., 2010); cadherin 2 (*CDH2*), which is involved in neuronal differentiation (Cherry et al., 2014); cysteine rich motor neuron 1 (*CRIM1*); neurexin (*NRXN1*); synuclein alpha (*SNCA*), which is a major component of amyloid plaques in patients with Alzheimers disease (Uéda et al., 1993; Matsubara et al., 2001; Lutz et al., 2015); and SRY-box 11 (*SOX11*), which plays a role in neural differentiation and the response to injury (Jankowski et al., 2006, 2009). *SNCA* and *SOX11* are differentially expressed both at the gene and CSI level along with stathmin 4 (*STMN4*), serotonin receptor 1B (*HTR1B*), and histone cluster 1 H2B family member G (*HIST1H2BG*). GO:BP enrichment of the DEGs resulted in a handful of categories generally related to neuronal dendrite development and synaptic transmission (Supplemental Figures 5–7; Supplemental Tables 4–6).

Our own rat dataset consists of a time series analysis of the transcriptional profile of the dorsal root ganglion (DRG) after sciatic nerve transection. For both differential gene expression and significantly changed CSIs, we focused on changes at an early (3 day) and late (90 day) time point after axotomy vs. untreated controls for the purpose of this study. At the early time point (day 3 vs. naïve), a large number of DEGs (1972) and genes with DE-CSIs using both the WITHIN (3,485) and DEXSeq (6,347) pipelines were detected (Table 6). By the late time point (day 90 vs. naïve), the number of DEGs (672) and genes with DE-CSIs (544 using WITHIN; 2,866 using DEXSeq) was greatly reduced.

In terms of the enriched biological processes for DE-CSIs, at day 3 vs. naïve, the top 25 GO terms for the DEXSeq CSI pipeline includes processes involved in axonogenesis (such as regulation of neuron projection development, axon development, axonogenesis, synapse organization, etc.) and transport (vacuolar transport, Golgi vesicle transport, endosomal transport, cytosolic transport, etc.). The WITHIN pipeline yields similar enriched processes.

At day 90 vs. naïve, the top enriched GO biological processes for the DEXSeq CSI pipeline can be grouped into processes involved in synapse formation (synapse assembly, dendrite development, regulation of neuron projection development, dendrite morphogenesis, regulation of synapse assembly, synapse organization, axonogenesis, gliogenesis, etc.) and muscle development (although in this context, more likely related to axon regeneration, since both contain actin filament organization, and actin filament polymerization). The results from the WITHIN CSI pipeline show a similar enrichment for synapse formation along with enrichments for cell morphogenesis (regulation of cell morphogenesis, regulation of cell morphogenesis involved in differentiation, etc.) and ion transport. These results indicate that the 3′UTR dynamics at day 3 appear involved with organizing transcripts for remodeling the damaged nerves, while at day 90, the function is shifted to reforming synapses.

A large number of significant events are found in common among all five time points (Supplemental Figure 8), including 260 CSI-related genes using the WITHIN pipeline, 677 using the DEXSeq pipeline, and 103 differentially expressed genes. Enrichment analysis for these overlapping events as determined by categoryCompare (Flight et al., 2014) (Supplemental Figure 9) yields 37 significant biological processes from the WITHIN pipeline, including those related to axon development, ion transport/synaptic transmission, organelle localization, and muscle contraction. Only six significant biological processes are enriched from the WITHIN pipeline, all related to dendrite development.

### Potential Mechanism for Alternative Polyadenylation

**Figure 6:**
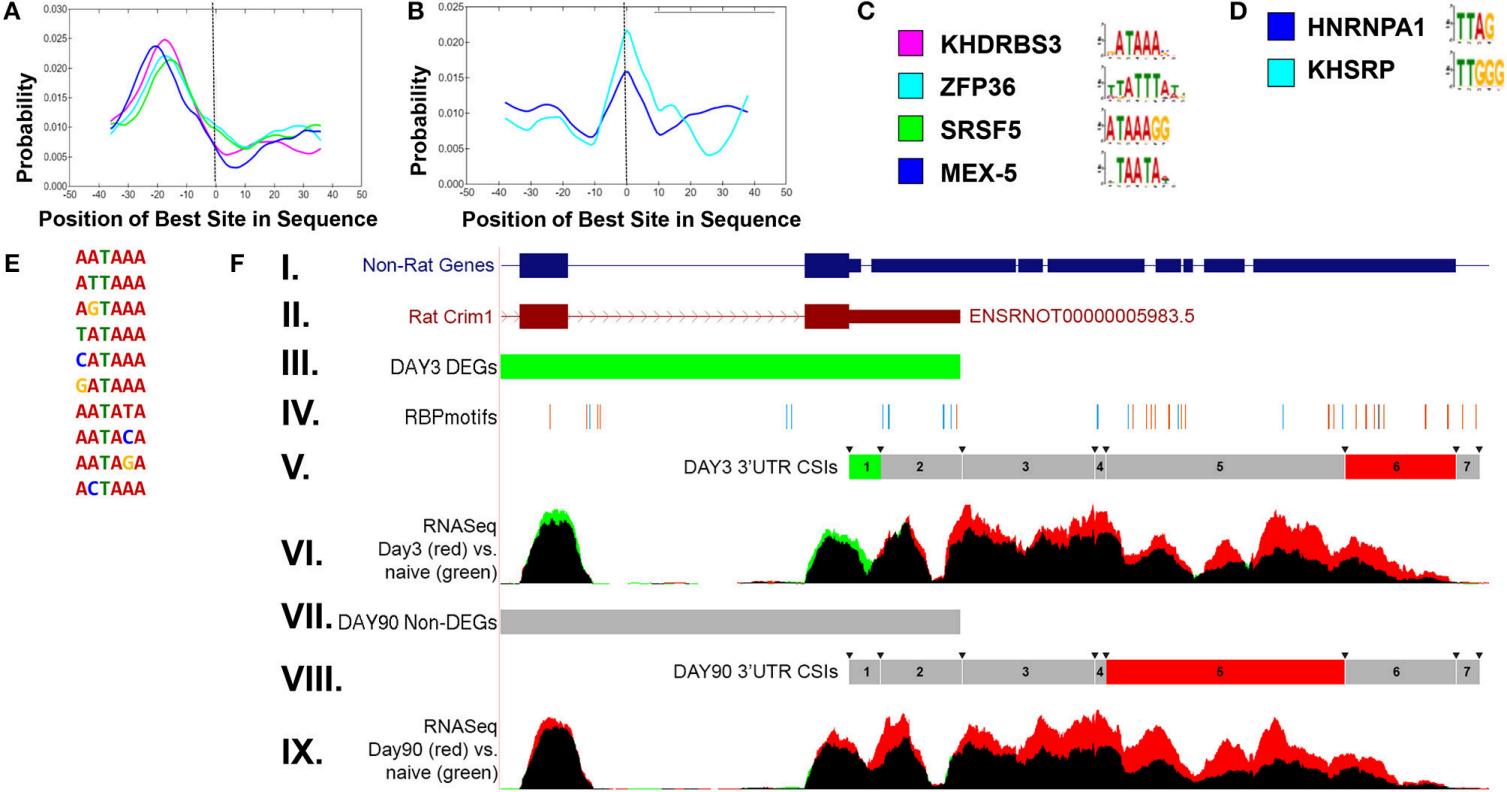
Enriched RNA binding motifs for differentially expressed CSIs in rat sciatic nerve transection. **(A)** shows enriched RNA binding protein (RBP) motifs *upstream* of significantly changed CSI sites. The location (−30 to −10) is consistent with previously reported results (Beaudoing et al., 2000). **(B)** shows enriched RBP motifs *overlapping* the boundaries of significantly changed CSI sites. The corresponding upstream and overlapping motif patterns are shown in **(C,D)**, respectively. The enriched upstream motifs **(A)** are consistent with the most prevalent polyadenylation sites **(E)**. **(F)** shows an example of the CSI-UTR analysis within the rat *Crim1* gene. In this case, the rat Crim1 UTR annotation (subpanel II) is significantly shortened when compared to *Crim1* homologs (subpanel I). Subpanels III and VII indicate the differential expression status at the gene level (using cuffdiff) for day 3 and day 90 vs. naïve, where red represents significant up-regulation, green represents significant down-regulation (as seen in day 3 vs. naïve, subpanel III) and gray represents a non-significant change (as seen in day 90 vs. naïve, subpanel VII). The location of RBP motifs from **(C,D)** are shown in subpanel IV, and tend to cluster around the CSI interval boundaries, which are indicated by the arrows in subpanels V and VIII. The CSI usage status for each of the seven *Crim1* CSIs is shown in subpanels V and VIII, where red represents a significant increase in utilization, green represents a significant decrease in utilization, and gray represents a non-significant difference. Using the information from subpanels V and VIII along with the mapped RNASeq reads in panels VI and IX, we can infer there are likely three separate 3′UTR isoforms present at both day 3 and day 90, with the short form terminating after CSI 2, the intermediate form terminating after CSI 4, and the long form terminating after CSI 6. In this case, there is not any sequence based evidence for extension of the 3′UTRs into CSI 7. These results for panel **(F)** indicate that in addition to the differential down regulation of *Crim1* at day 3, that *Crim1* isoforms are typically elongated in the UTR at both experimental day 3 and day 90 when compared to naïve.

In order to examine potential mechanisms for condition-dependent and tissue-specific alternative polyadenylation, RNA-binding protein (RBP) motif enrichment was performed using the MEME suite (Bailey et al., 2015). The results indicate an enrichment of RBP motifs near the polyA signal, −30 to −10 bp relative to the CSI site, as well as overlapping the junction between two adjacent CSIs. Figure 6 shows representative RBP enrichment results for differentially expressed CSIs at 90 days post sciatic crush. Included are a set of RBPs (*KHRRBS3, ZFP36, SRS5*, and *MEX-5*) (Figure 6A) with motifs enriched near the polyA signal, and a set of RBP motifs overlapping the CSI junction, including *HNRNP-A1* and *KHSRP* (Figure 6B). Consensus motifs for each of the sets are provided in Figures 6C,D. The motifs found near the polyA signal have consensus patterns overlapping the most common functional polyA signals (Figure 6E) (Beaudoing et al., 2000). The RBP *HNRNP-A1* overlapping CSI junctions is of particular interest, due to its multiplicity of roles in mRNA processing (Jean-Philippe et al., 2013), suggesting it could potentially play a role in alternative polyadenylation. A specific enrichment example is shown in Figure 6F for *CRIM1* (cysteine-rich motor neuron 1) within the rat sciatic transection models at both day 3 and day 90. This illustrates an enrichment of motifs within the ends of the CSIs, consistent with the overall patterns in panels A-D. *CRIM1* is determined to have significant alternative polyadenylation at both day 3 and day 90, suggesting lengthened UTRs compared to naïve. However, the gene is significantly down-regulated at day 3, as represented by the green values in the final two exons represented in Figure 6F. We often observe the combination of these two events appear to cancel each other out, resulting in insignificant differences at the gene level, suggesting the importance of separating the coding region from the UTR in differential expression analysis. In addition, the case of the rat *CRIM1* gene shows the issue with reliance of annotations in the UTRs, since the annotated rat 3′ UTR ends after the second CSI (top of Figure 6F) while RNAseq and non-rat models extend the 3′ UTR by approximately 2000 bases.

A similar pattern of RBP is found in the mouse optic nerve crush (ONC) model where *HNRNP-A1* is enriched across the CSI junctions (Supplemental Figure 10). An ONC-specific set of motifs overlapping the CSI junctions was found, including *PFF0320C, CG2931, NOVA1, SXL*, and *HEN1*. *CRIM1* also shows differential expression of the UTRs in the ONC model, but is not shown to be differentially expressed at the gene level. However, as can be seen in the RNA-Seq reads mapped in Supplemental Figure 10C, this gene is likely up-regulated in optic nerve crush (red) with a shortened 3′UTR (green). The two events work in concert to cancel each other out, resulting in insignificant p-values.

## Discussion

### Existing Computational Methods for Alternative Polyadenylation Detection

In addition to our CSI-UTR approach, a number of methodologies have recently appeared to measure alternative polyadenylation events. Many of these have been previously reviewed (Yeh et al., 2017). A comparison of these approaches is provided in Table 7. Most of these attempt to detect a difference between a short and long form UTR (Wang et al., 2014; Shenker et al., 2015; Grassi et al., 2016). However, such an approach is insensitive to the presence of three or more APA events. To address this, KLEAT (Birol et al., 2015) attempts to characterize cleavage sites using polyA sequencing data, but their system does not analyze differential expression within the resulting intervals. The two approaches most closely related to CSI-UTR are GETUTR (Kim M. et al., 2015) and DaPars (Xia et al., 2014). GETUTR performs the step of estimating the 3′ UTR landscape from RNASeq data using heuristic and regression methods. However, while GETUTR detects likely events, it does not appear to determine the significance of these events, and thus is comparable to our initial detection of CSIs. In addition, GETUTR is limited since it only allows for analysis of the human genome, specifically the hg19 assembly. DaPars is the closest computational approach to CSI-UTR. It functions by computing a usage difference between distal and proximal APAs in two conditions, using individual replicates in a pairwise fashion with a Fisher's exact test, and is able to detect multiple APA events. The main differences between DaPars and CSI-UTR is the approach to detecting changes. CSI-UTR considers individual CSIs and their differential expression while DaPars considers the relationship between a proximal and distal APA site in a pairwise fashion. As a result, CSI-UTR offers a greater ability to localize interval regions where changes occur in the UTR, thereby allowing for greater examination of functional motifs within these regions.

**Table 7:** Comparison of alternative polyadenylation approaches.

**Method**	**Regions reported**	**Genomes supported**	**Requires UTR annotation**	**Performs UTR differential expression**
CSI-UTR	Cleavage site intervals	hg38, mm10, rn6 Others with CSI BED file	No	Yes
DaPars	Tandem APA sites	Any with gene BED file	No	Yes
Roar	Tandem APA sites	hg19, mm9	Yes	Yes
IsoSCM	Tandem APA sites	Any with aligned BAM file	No	No
3USS	Tandem APA sites	hg19, mm9, rn4, bosTau4, canFam2, Galgal3, dm3, ce10	No	No
KLEAT	Identifies polyA cleavage sites	Any with aligned BAM file and gene GTF file	No	No
GETUTR	Identifies polyA cleavage sites	hg19	No	No

### Performance Comparison of CSI-UTR to DaPars

We compared CSI-UTR using both the WITHIN and DEXSeq methods to the most closely related method, DaPars (Supplemental Figure 11). For the human LOAD experiment, the WITHIN method yields 912 Ensembl genes with significantly differentially expressed CSIs that are not found by either DEXSeq or DaPars; 17 Ensembl genes are found to have differentially expressed CSIs only by DEXSeq; and 265 genes found with alternative polyadenylation sites only within DaPars. Since both the WITHIN and DaPars methods collapse replicate information, both will be less susceptible to individual sample variation, unlike the DEXSeq method. Thus, both are likely to increase both true positive and false positive sample rates. Further examination of the 912 genes with differentially expressed CSIs indicates that 900 have 3 or more CSIs, and are thus unlikely to be identified with DaPars which only accounts for short and long UTR forms. Of the remaining twelve, eight appear to be true positives based on RNA-Seq evidence, while four appear to be false positives due to previously unannotated transcripts appearing within the region identified as a CSI. Examination of the 265 genes with APA events found only by DaPars indicates that 202 of these have alternative stop codons. These are likely to be missed by CSI-UTR because only the distal-most stop codon is used, since any internal reads between stop codons may result from either coding exons or untranslated exons, depending upon the specific transcript. Thus, reads in these regions can potentially indicate either alternative coding exon usage, or alternative polyadenylation, neither of which can be easily inferred from RNA-Seq data alone. For the remaining sequences, 56 only have a single CSI identified within their 3′ UTR, indicating that there is not any evidence of polyadenylation from the poly-A seq data of Derti et al. (2012). Seven of the remaining sequences appear to be false positives due to variability in the sample sequences, while one of the sequences has multiple UTR isoforms present, and is likely a true positive that is missed by the other methods due to the absence of polyA-Seq data for this transcript. Further analysis of the seventeen genes found to have differential CSIs only with the DEXSeq method shows that 16 of these have 3 or more CSIs, and in each case, the overall expression levels are low, as is the variability between the samples.

### CSI-UTR Benefits

Analysis of the coding regions of transcripts, both in terms of differential expression and to a lesser degree alternative splicing, has achieved a level of standardization such that it is largely accurate, useful, and broadly-approachable. However, this is not true of UTR-related events, and our results demonstrate that additional biological control mechanisms can be uncovered by considering the dynamics of the 3′ UTR. This is important because many of these UTR events act independently of the coding region, as demonstrated in our earlier work on *CAMK4* (Harrison et al., 2014). One of the benefits of high-throughput sequencing over array technologies is the ability to measure everything that is transcribed, including the untranslated regions. Therefore, we are able to utilize publicly available datasets by extending our analysis outside of the CDS and into the UTR. This allows for the detection of additional events occurring within the UTR region of transcripts, which are enriched within the central and peripheral nervous systems, in cancer, and during development. Studies examining timing mechanisms and involving localization of transcripts are likely to benefit most from our approaches due to changes associated with transcript stability and subcellular localization that can be controlled by interactions within the 3′ UTR. Even in cases where differential gene expression is minimal, it is possible a biological process will be regulated by these alterations in 3′ UTRs. Given the set of differentially expressed (upregulated or downregulated) CSIs, the next step in analysis was to determine functional domains included or excluded, as we have previously done for alternative splicing events (Park et al., 2016). Patterns likely to be elicited include miRNA binding sites, RNA binding protein sites, and additional motifs that could impact on patterns of CSI usage. The results of the RBP motif enrichment near the polyA signal and the CSI junction site indicate potential mechanisms involving specific proteins, including: *KHRRBS3, ZFP36, SRS5, MEX-5, HNRNP-A1, KHSRP, PFF0320C, CG2931, NOVA1, SXL*, and *HEN1* for condition- and tissue-specific alternative polyadenylation. Further analyses of these 3′UTR RNA binding partners will hopefully prove beneficial in understanding alternative polyadenylation mechanisms.

### Limitations of CSI-UTR

The main limitation of CSI-UTR is the reliance on polyA-Seq data for the construction of cleavage site intervals. It is possible therefore that some of the APA events will be missed. However, as more comprehensive polyA sequencing data becomes available, covering a multitude of tissue types, developmental stages, and phenotypic conditions, the more complete the detection of APA events CSI-UTR will be able to detect. In addition, CSI-UTR currently focuses on coding mRNAs due to the necessity of finding the distal-most stop codon. However, this approach may miss some CSIs occurring due to alternative stop codons and will also limit the detection of APAs in non-coding genes, such as lncRNAs, which may be polyadenylated as well.

## Conclusion

The method presented here, CSI-UTR, allows for: (1) the detection of cleavage site intervals between the stop codon and the 3′ terminal end, while (2) detecting differential usage of alternative polyadenylation sites. Such an approach enables the analysis of 3′ UTR dynamics in a specific experimental condition. Our initial results based on publicly available datasets highlight the potential benefit of further utilizing these datasets, offering additional insight into processes involving the 3′ UTR, including cellular localization, regulation of translational control mechanisms, and transcript stability.

## Author Contributions

BH, JCP, and ER contributed conception and design of the CSI-UTR algorithm. BH and ER implemented the algorithm. JWP, CG, and JC provided analysis of RNA binding enrichment and Genome Browser tracks. BH, JCP, and CG contributed to the analysis of the mouse and human datasets. MS and MI provided the rat dataset and its subsequent analysis. BH and ER wrote the first draft of the manuscript. All authors contributed to manuscript revision, read and approved the submitted version.

### Conflict of Interest Statement

The authors declare that the research was conducted in the absence of any commercial or financial relationships that could be construed as a potential conflict of interest.

## References

[B1] Akman H. B. Oyken M. Tuncer T. Can T. Erson-Bensan A. E. (2015). 3'UTR shortening and EGF signaling: implications for breast cancer. Hum. Mol. Genet. 24, 6910–6920. 10.1093/hmg/ddv391 26395459

[B2] Alonso Vilatela M. E. Lopez-Lopez M. Yescas-Gomez P. (2012). Genetics of Alzheimer's disease. Arch. Med. Res. 43, 622–631. 10.1016/j.arcmed.2012.10.017 23142261

[B3] An J. J. Gharami K. Liao G. Y. Woo N. H. Lau A. G. Vanevski F.. (2008). Distinct role of long 3' UTR BDNF mRNA in spine morphology and synaptic plasticity in hippocampal neurons. Cell 134, 175–187. 10.1016/j.cell.2008.05.045 18614020 PMC2527207

[B4] Anders S. Reyes A. Huber W. (2012). Detecting differential usage of exons from RNA-seq data. Genome Res. 22, 2008–2017. 10.1101/gr.133744.111 22722343 PMC3460195

[B5] Arefeen A. Liu J. Xiao X. Jiang T. (2018). TAPAS: tool for alternative polyadenylation site analysis. Bioinformatics 34, 2521–2529. 10.1093/bioinformatics/bty110 30052912 PMC6454472

[B6] Aschrafi A. Natera-Naranjo O. Gioio A. E. Kaplan B. B. (2010). Regulation of axonal trafficking of cytochrome c oxidase IV mRNA. Mol. Cell. Neurosci. 43, 422–430. 10.1016/j.mcn.2010.01.009 20144716 PMC2845174

[B7] Bailey T. L. Johnson J. Grant C. E. Noble W. S. (2015). The MEME Suite. Nucleic Acids Res. 43, W39–W49. 10.1093/nar/gkv416 25953851 PMC4489269

[B8] Beaudoing E. Freier S. Wyatt J. R. Claverie J. M. Gautheret D. (2000). Patterns of variant polyadenylation signal usage in human genes. Genome Res. 10, 1001–1010. 10.1101/gr.10.7.1001 10899149 PMC310884

[B9] Bienroth S. Keller W. Wahle E. (1993). Assembly of a processive messenger RNA polyadenylation complex. EMBO J. 12, 585–594. 10.1002/j.1460-2075.1993.tb05690.x 8440247 PMC413241

[B10] Birol I. Raymond A. Chiu R. Nip K. M. Jackman S. D. Kreitzman M. (2015). Kleat: cleavage site analysis of transcriptomes. Pac. Symp. Biocomput. 2015, 347–358. 10.1142/9789814644730_0034 PMC4350765 25592595

[B11] Bitto E. Bingman C. A. Wesenberg G. E. Mccoy J. G. Phillips G. N. Jr. (2007). Structure of aspartoacylase, the brain enzyme impaired in Canavan disease. Proc. Natl. Acad. Sci. U.S.A. 104, 456–461. 10.1073/pnas.0607817104 17194761 PMC1766406

[B12] Blichenberg A. Schwanke B. Rehbein M. Garner C. C. Richter D. Kindler S. (1999). Identification of a cis-acting dendritic targeting element in MAP2 mRNAs. J. Neurosci. 19, 8818–8829. 10.1523/JNEUROSCI.19-20-08818.1999 10516301 PMC6782761

[B13] Böckers T. M. Segger-Junius M. Iglauer P. Bockmann J. Gundelfinger E. D. Kreutz M. R.. (2004). Differential expression and dendritic transcript localization of Shank family members: identification of a dendritic targeting element in the 3' untranslated region of Shank1 mRNA. Mol. Cell. Neurosci. 26, 182–190. 10.1016/j.mcn.2004.01.009 15121189

[B14] Bolger A. M. Lohse M. Usadel B. (2014). Trimmomatic: a flexible trimmer for Illumina sequence data. Bioinformatics 30, 2114–2120. 10.1093/bioinformatics/btu170 24695404 PMC4103590

[B15] Carrington J. C. Ambros V. (2003). Role of microRNAs in plant and animal development. Science 301, 336–338. 10.1126/science.1085242 12869753

[B16] Chen C. Y. Shyu A. B. (1995). AU-rich elements: characterization and importance in mRNA degradation. Trends Biochem. Sci. 20, 465–470. 10.1016/S0968-0004(00)89102-1 8578590

[B17] Cherry J. F. Bennett N. K. Schachner M. Moghe P. V. (2014). Engineered N-cadherin and L1 biomimetic substrates concertedly promote neuronal differentiation, neurite extension and neuroprotection of human neural stem cells. Acta Biomater. 10, 4113–4126. 10.1016/j.actbio.2014.06.001 24914828

[B18] Consortium S. M.-I. (2014). A comprehensive assessment of RNA-seq accuracy, reproducibility and information content by the sequencing quality control consortium. Nat. Biotechnol. 32, 903–914. 10.1038/nbt.2957 25150838 PMC4321899

[B19] de Sauvage F. Kruys V. Marinx O. Huez G. Octave J. N. (1992). Alternative polyadenylation of the amyloid protein precursor mRNA regulates translation. EMBO J. 11, 3099–3103. 10.1002/j.1460-2075.1992.tb05382.x 1353447 PMC556794

[B20] Derti A. Garrett-Engele P. Macisaac K. D. Stevens R. C. Sriram S. Chen R.. (2012). A quantitative atlas of polyadenylation in five mammals. Genome Res. 22, 1173–1183. 10.1101/gr.132563.111 22454233 PMC3371698

[B21] Deuel T. A. Liu J. S. Corbo J. C. Yoo S. Y. Rorke-Adams L. B. Walsh C. A. (2006). Genetic interactions between doublecortin and doublecortin-like kinase in neuronal migration and axon outgrowth. Neuron 49, 41–53. 10.1016/j.neuron.2005.10.038 16387638

[B22] Di Giammartino D. C. Nishida K. Manley J. L. (2011). Mechanisms and consequences of alternative polyadenylation. Mol. Cell 43, 853–866. 10.1016/j.molcel.2011.08.017 21925375 PMC3194005

[B23] Dickson J. R. Kruse C. Montagna D. R. Finsen B. Wolfe M. S. (2013). Alternative polyadenylation and miR-34 family members regulate tau expression. J. Neurochem. 127, 739–749. 10.1111/jnc.12437 24032460 PMC3859707

[B24] Dobin A. Davis C. A. Schlesinger F. Drenkow J. Zaleski C. Jha S.. (2013). STAR: ultrafast universal RNA-seq aligner. Bioinformatics 29, 15–21. 10.1093/bioinformatics/bts635 23104886 PMC3530905

[B25] Flight R. M. Harrison B. J. Mohammad F. Bunge M. B. Moon L. D. Petruska J. C.. (2014). categoryCompare, an analytical tool based on feature annotations. Front. Genet. 5:98. 10.3389/fgene.2014.00098 24808906 PMC4010757

[B26] Flynn R. A. Almada A. E. Zamudio J. R. Sharp P. A. (2011). Antisense RNA polymerase II divergent transcripts are P-TEFb dependent and substrates for the RNA exosome. Proc. Natl. Acad. Sci. U.S.A. 108, 10460–10465. 10.1073/pnas.1106630108 21670248 PMC3127934

[B27] Fox-Walsh K. Davis-Turak J. Zhou Y. Li H. Fu X. D. (2011). A multiplex RNA-seq strategy to profile poly(A+) RNA: application to analysis of transcription response and 3' end formation. Genomics 98, 266–271. 10.1016/j.ygeno.2011.04.003 21515359 PMC3160523

[B28] Fu Y. Sun Y. Li Y. Li J. Rao X. Chen C.. (2011). Differential genome-wide profiling of tandem 3' UTRs among human breast cancer and normal cells by high-throughput sequencing. Genome Res. 21, 741–747. 10.1101/gr.115295.110 21474764 PMC3083091

[B29] Giudice G. Sánchez-Cabo F. Torroja C. Lara-Pezzi E. (2016). ATtRACT-a database of RNA-binding proteins and associated motifs. Database (Oxford) 2016:baw035. 10.1093/database/baw035 27055826 PMC4823821

[B30] Grassi E. Mariella E. Lembo A. Molineris I. Provero P. (2016). Roar: detecting alternative polyadenylation with standard mRNA sequencing libraries. BMC Bioinformatics 17:423. 10.1186/s12859-016-1254-8 27756200 PMC5069797

[B31] Grupe A. Li Y. Rowland C. Nowotny P. Hinrichs A. L. Smemo S.. (2006). A scan of chromosome 10 identifies a novel locus showing strong association with late-onset Alzheimer disease. Am. J. Hum. Genet. 78, 78–88. 10.1086/498851 16385451 PMC1380225

[B32] Guan Z. Kuhn J. A. Wang X. Colquitt B. Solorzano C. Vaman S.. (2016). Injured sensory neuron-derived CSF1 induces microglial proliferation and DAP12-dependent pain. Nat. Neurosci. 19, 94–101. 10.1038/nn.4189 26642091 PMC4703328

[B33] Ha K. C. H. Blencowe B. J. Morris Q. (2018). QAPA: a new method for the systematic analysis of alternative polyadenylation from RNA-seq data. Genome Biol. 19:45. 10.1186/s13059-018-1414-4 29592814 PMC5874996

[B34] Harrison B. J. Flight R. M. Gomes C. Venkat G. Ellis S. R. Sankar U.. (2014). IB4-binding sensory neurons in the adult rat express a novel 3' UTR-extended isoform of CaMK4 that is associated with its localization to axons. J. Comput. Neurol. 522, 308–336. 10.1002/cne.23398 23817991 PMC3855891

[B35] Hartley S. W. Mullikin J. C. (2016). Detection and visualization of differential splicing in RNA-Seq data with JunctionSeq. Nucleic Acids Res. 44:e127. 10.1093/nar/gkw501 27257077 PMC5009739

[B36] Hilgers V. Perry M. W. Hendrix D. Stark A. Levine M. Haley B. (2011). Neural-specific elongation of 3′ UTRs during *Drosophila* development. Proc. Natl. Acad. Sci.U.S.A. 108, 15864–15869. 10.1073/pnas.1112672108 21896737 PMC3179109

[B37] Hochberg Y. Benjamini Y. (1990). More powerful procedures for multiple significance testing. Stat. Med. 9, 811–818. 10.1002/sim.4780090710 2218183

[B38] Howe K. L. Bolt B. J. Cain S. Chan J. Chen W. J. Davis P.. (2016). WormBase 2016: expanding to enable helminth genomic research. Nucleic Acids Res. 44, D774–D780. 10.1093/nar/gkv1217 26578572 PMC4702863

[B39] Hu Y. Huang Y. Du Y. Orellana C. F. Singh D. Johnson A. R.. (2013). DiffSplice: the genome-wide detection of differential splicing events with RNA-seq. Nucleic Acids Res. 41:e39. 10.1093/nar/gks1026 23155066 PMC3553996

[B40] Jankowski M. P. Cornuet P. K. Mcilwrath S. Koerber H. R. Albers K. M. (2006). SRY-box containing gene 11 (Sox11) transcription factor is required for neuron survival and neurite growth. Neuroscience 143, 501–514. 10.1016/j.neuroscience.2006.09.010 17055661 PMC1698553

[B41] Jankowski M. P. Mcilwrath S. L. Jing X. Cornuet P. K. Salerno K. M. Koerber H. R.. (2009). Sox11 transcription factor modulates peripheral nerve regeneration in adult mice. Brain Res. 1256, 43–54. 10.1016/j.brainres.2008.12.032 19133245 PMC2666926

[B42] Jansen R. P. (2001). mRNA localization: message on the move. Nat. Rev. Mol. Cell Biol. 2, 247–256. 10.1038/35067016 11283722

[B43] Jean-Philippe J. Paz S. Caputi M. (2013). hnRNP A1: the Swiss army knife of gene expression. Int. J. Mol. Sci. 14, 18999–19024. 10.3390/ijms140918999 24065100 PMC3794818

[B44] Ji G. Guan J. Zeng Y. Li Q. Q. Wu X. (2015). Genome-wide identification and predictive modeling of polyadenylation sites in eukaryotes. Brief. Bioinform. 16, 304–313. 10.1093/bib/bbu011 24695098

[B45] Ji Z. Lee J. Y. Pan Z. Jiang B. Tian B. (2009). Progressive lengthening of 3′ untranslated regions of mRNAs by alternative polyadenylation during mouse embryonic development. Proc. Natl. Acad. Sci.U.S.A. 106, 7028–7033. 10.1073/pnas.0900028106 19372383 PMC2669788

[B46] Katz Y. Wang E. T. Airoldi E. M. Burge C. B. (2010). Analysis and design of RNA sequencing experiments for identifying isoform regulation. Nat. Methods 7, 1009–1015. 10.1038/nmeth.1528 21057496 PMC3037023

[B47] Kent W. J. Sugnet C. W. Furey T. S. Roskin K. M. Pringle T. H. Zahler A. M.. (2002). The human genome browser at UCSC. Genome Res. 12, 996–1006. 10.1101/gr.229102 12045153 PMC186604

[B48] Kim D. Langmead B. Salzberg S. L. (2015). HISAT: a fast spliced aligner with low memory requirements. Nat. Methods 12, 357–360. 10.1038/nmeth.3317 25751142 PMC4655817

[B49] Kim D. Pertea G. Trapnell C. Pimentel H. Kelley R. Salzberg S. L. (2013). TopHat2: accurate alignment of transcriptomes in the presence of insertions, deletions and gene fusions. Genome Biol. 14:R36. 10.1186/gb-2013-14-4-r36 23618408 PMC4053844

[B50] Kim M. You B. H. Nam J. W. (2015). Global estimation of the 3' untranslated region landscape using RNA sequencing. Methods 83, 111–117. 10.1016/j.ymeth.2015.04.011 25899044

[B51] Kislauskis E. H. Zhu X. Singer R. H. (1994). Sequences responsible for intracellular localization of beta-actin messenger RNA also affect cell phenotype. J. Cell Biol. 127, 441–451. 10.1083/jcb.127.2.441 7929587 PMC2120214

[B52] Kobayashi H. Yamamoto S. Maruo T. Murakami F. (2005). Identification of a cis-acting element required for dendritic targeting of activity-regulated cytoskeleton-associated protein mRNA. Eur. J. Neurosci. 22, 2977–2984. 10.1111/j.1460-9568.2005.04508.x 16367764

[B53] Kuersten S. Goodwin E. B. (2003). The power of the 3[prime] UTR: translational control and development. Nat. Rev. Genet. 4, 626–637. 10.1038/nrg1125 12897774

[B54] Latour P. Thauvin-Robinet C. Baudelet-Méry C. Soichot P. Cusin V. Faivre L.. (2010). A major determinant for binding and aminoacylation of tRNA(Ala) in cytoplasmic Alanyl-tRNA synthetase is mutated in dominant axonal charcot-marie-tooth disease. Am. J. Hum. Genet. 86, 77–82. 10.1016/j.ajhg.2009.12.005 20045102 PMC2801750

[B55] Le Pera L. Mazzapioda M. Tramontano A. (2015). 3USS: a web server for detecting alternative 3'UTRs from RNA-seq experiments. Bioinformatics 31, 1845–1847. 10.1093/bioinformatics/btv035 25617413 PMC4443675

[B56] Leinonen R. Sugawara H. Shumway M. International Nucleotide Sequence Database C. (2011). The sequence read archive. Nucleic Acids Res. 39, D19–D21. 10.1093/nar/gkq1019 21062823 PMC3013647

[B57] Lembo A. Di Cunto F. Provero P. (2012). Shortening of 3′UTRs correlates with poor prognosis in breast and lung cancer. PLoS ONE 7:e31129. 10.1371/journal.pone.0031129 22347440 PMC3275581

[B58] Leube R. E. (1994). Expression of the synaptophysin gene family is not restricted to neuronal and neuroendocrine differentiation in rat and human. Differentiation 56, 163–171. 10.1046/j.1432-0436.1994.5630163.x 8034131

[B59] Li H. Handsaker B. Wysoker A. Fennell T. Ruan J. Homer N.. (2009). The sequence alignment/map format and SAMtools. Bioinformatics 25, 2078–2079. 10.1093/bioinformatics/btp352 19505943 PMC2723002

[B60] Li J. Li R. You L. Xu A. Fu Y. Huang S. (2015). Evaluation of two statistical methods provides insights into the complex patterns of alternative polyadenylation site switching. PLoS ONE 10:e0124324. 10.1371/journal.pone.0124324 25875641 PMC4396989

[B61] Li Z. Mulligan M. K. Wang X. Miles M. F. Lu L. Williams R. W. (2010). A transposon in Comt generates mRNA variants and causes widespread expression and behavioral differences among mice. PLoS ONE 5:e12181. 10.1371/journal.pone.0012181 20808911 PMC2923157

[B62] Love J. E. Hayden E. J. Rohn T. T. (2015). Alternative splicing in Alzheimer's disease. J. Parkinsons Dis. Alzheimers Dis. 2:6. 10.13188/2376-922X.1000010 26942228 PMC4772657

[B63] Love M. I. Huber W. Anders S. (2014). Moderated estimation of fold change and dispersion for RNA-seq data with DESeq2. Genome Biol. 15:550. 10.1186/s13059-014-0550-8 25516281 PMC4302049

[B64] Lu J. Bushel P. R. (2013). Dynamic expression of 3' UTRs revealed by poisson hidden markov modeling of RNA-Seq: implications in gene expression profiling. Gene 527, 616–623. 10.1016/j.gene.2013.06.052 23845781 PMC3902974

[B65] Lutz M. W. Saul R. Linnertz C. Glenn O. C. Roses A. D. Chiba-Falek O. (2015). A cytosine-thymine (CT)-rich haplotype in intron 4 of SNCA confers risk for Lewy body pathology in Alzheimer's disease and affects SNCA expression. Alzheimers Dement. 11, 1133–1143. 10.1016/j.jalz.2015.05.011 26079410 PMC4630109

[B66] Mangone M. Manoharan A. P. Thierry-Mieg D. Thierry-Mieg J. Han T. Mackowiak S. D.. (2010). The landscape of C. elegans 3'UTRs. Science 329, 432–435. 10.1126/science.1191244 20522740 PMC3142571

[B67] Matsubara M. Yamagata H. Kamino K. Nomura T. Kohara K. Kondo I.. (2001). Genetic association between Alzheimer disease and the alpha-synuclein gene. Dement. Geriatr. Cogn. Disord. 12, 106–109. 10.1159/000051243 11173882

[B68] Mayr C. Bartel D. P. (2009). Widespread shortening of 3'UTRs by alternative cleavage and polyadenylation activates oncogenes in cancer cells. Cell 138, 673–684. 10.1016/j.cell.2009.06.016 19703394 PMC2819821

[B69] Mcgrew L. L. Dworkin-Rastl E. Dworkin M. B. Richter J. D. (1989). Poly(A) elongation during Xenopus oocyte maturation is required for translational recruitment and is mediated by a short sequence element. Genes Dev. 3, 803–815. 10.1101/gad.3.6.803 2568313

[B70] Meer E. J. Wang D. O. Kim S. Barr I. Guo F. Martin K. C. (2012). Identification of a cis-acting element that localizes mRNA to synapses. Proc. Natl. Acad. Sci. U.S.A. 109, 4639–4644. 10.1073/pnas.1116269109 22383561 PMC3311331

[B71] Mercer T. R. Gerhardt D. J. Dinger M. E. Crawford J. Trapnell C. Jeddeloh J. A.. (2012). Targeted RNA sequencing reveals the deep complexity of the human transcriptome. Nat. Biotechnol. 30, 99–104. 10.1038/nbt.2024 22081020 PMC3710462

[B72] Mercer T. R. Wilhelm D. Dinger M. E. Sold,à G. Korbie D. J. Glazov E. A.. (2011). Expression of distinct RNAs from 3′ untranslated regions. Nucleic Acids Res. 39, 2393–2403. 10.1093/nar/gkq1158 21075793 PMC3064787

[B73] Merianda T. T. Coleman J. Kim H. H. Kumar Sahoo P. Gomes C. Brito-Vargas P.. (2015). Axonal amphoterin mRNA is regulated by translational control and enhances axon outgrowth. J. Neurosci. 35, 5693–5706. 10.1523/JNEUROSCI.3397-14.2015 25855182 PMC4388927

[B74] Mori Y. Imaizumi K. Katayama T. Yoneda T. Tohyama M. (2000). Two cis-acting elements in the 3' untranslated region of alpha-CaMKII regulate its dendritic targeting. Nat. Neurosci. 3, 1079–1084. 10.1038/80591 11036263

[B75] O'leary N. A. Wright M. W. Brister J. R. Ciufo S. Haddad D. Mcveigh R.. (2016). Reference sequence (RefSeq) database at NCBI: current status, taxonomic expansion, and functional annotation. Nucleic Acids Res. 44, D733–D745. 10.1093/nar/gkv1189 26553804 PMC4702849

[B76] Park J. W. Jung S. Rouchka E. C. Tseng Y. T. Xing Y. (2016). rMAPS: RNA map analysis and plotting server for alternative exon regulation. Nucleic Acids Res. 44, W333–W338. 10.1093/nar/gkw410 27174931 PMC4987942

[B77] Patel V. L. Mitra S. Harris R. Buxbaum A. R. Lionnet T. Brenowitz M.. (2012). Spatial arrangement of an RNA zipcode identifies mRNAs under post-transcriptional control. Genes Dev. 26, 43–53. 10.1101/gad.177428.111 22215810 PMC3258965

[B78] Prakash N. Fehr S. Mohr E. Richter D. (1997). Dendritic localization of rat vasopressin mRNA: ultrastructural analysis and mapping of targeting elements. Eur. J. Neurosci. 9, 523–532. 10.1111/j.1460-9568.1997.tb01629.x 9104594

[B79] Quinlan A. R. (2014). BEDTools: the Swiss-army tool for genome feature analysis. Curr. Protoc. Bioinformatics 47, 11–34. 10.1002/0471250953.bi1112s47 25199790 PMC4213956

[B80] Quinlan A. R. Hall I. M. (2010). BEDTools: a flexible suite of utilities for comparing genomic features. Bioinformatics 26, 841–842. 10.1093/bioinformatics/btq033 20110278 PMC2832824

[B81] Raju C. S. Fukuda N. López-Iglesias C. Göritz C. Visa N. Percipalle P. (2011). In neurons, activity-dependent association of dendritically transported mRNA transcripts with the transacting factor CBF-A is mediated by A2RE/RTS elements. Mol. Biol. Cell 22, 1864–1877. 10.1091/mbc.e10-11-0904 21471000 PMC3103402

[B82] Sandberg R. Neilson J. R. Sarma A. Sharp P. A. Burge C. B. (2008). Proliferating cells express mRNAs with shortened 3' untranslated regions and fewer microRNA target sites. Science 320, 1643–1647. 10.1126/science.1155390 18566288 PMC2587246

[B83] Shaw G. Kamen R. (1986). A conserved AU sequence from the 3' untranslated region of GM-CSF mRNA mediates selective mRNA degradation. Cell 46, 659–667. 10.1016/0092-8674(86)90341-7 3488815

[B84] Shen S. Park J. W. Huang J. Dittmar K. A. Lu Z. X. Zhou Q.. (2012). MATS: a Bayesian framework for flexible detection of differential alternative splicing from RNA-seq data. Nucleic Acids Res. 40:e61. 10.1093/nar/gkr1291 22266656 PMC3333886

[B85] Shen S. Park J. W. Lu Z. X. Lin L. Henry M. D. Wu Y. N.. (2014). rMATS: robust and flexible detection of differential alternative splicing from replicate RNA-seq data. Proc. Natl. Acad. Sci. U.S.A. 111, E5593–E5601. 10.1073/pnas.1419161111 25480548 PMC4280593

[B86] Shenker S. Miura P. Sanfilippo P. Lai E. C. (2015). IsoSCM: improved and alternative 3' UTR annotation using multiple change-point inference. RNA 21, 14–27. 10.1261/rna.046037.114 25406361 PMC4274634

[B87] Shepard P. J. Choi E. A. Lu J. Flanagan L. A. Hertel K. J. Shi Y. (2011). Complex and dynamic landscape of RNA polyadenylation revealed by PAS-seq. RNA 17, 761–772. 10.1261/rna.2581711 21343387 PMC3062186

[B88] Sheppard S. Lawson N. D. Zhu L. J. (2013). Accurate identification of polyadenylation sites from 3' end deep sequencing using a naive Bayes classifier. Bioinformatics 29, 2564–2571. 10.1093/bioinformatics/btt446 23962617 PMC3789547

[B89] Subramanian M. Rage F. Tabet R. Flatter E. Mandel J. L. Moine H. (2011). G–quadruplex RNA structure as a signal for neurite mRNA targeting. EMBO Rep. 12, 697–704. 10.1038/embor.2011.76 21566646 PMC3128965

[B90] Szkop K. J. Nobeli I. (2017). Untranslated parts of genes interpreted: making heads or tails of high-throughput transcriptomic data via computational methods: computational methods to discover and quantify isoforms with alternative untranslated regions. Bioessays 39:1700090. 10.1002/bies.201700090 29052251

[B91] Tian B. Hu J. Zhang H. Lutz C. S. (2005). A large-scale analysis of mRNA polyadenylation of human and mouse genes. Nucleic Acids Res. 33, 201–212. 10.1093/nar/gki158 15647503 PMC546146

[B92] Uéda K. Fukushima H. Masliah E. Xia Y. Iwai A. Yoshimoto M.. (1993). Molecular cloning of cDNA encoding an unrecognized component of amyloid in Alzheimer disease. Proc. Natl. Acad. Sci. U.S.A. 90, 11282–11286. 10.1073/pnas.90.23.11282 8248242 PMC47966

[B93] Wang K. Singh D. Zeng Z. Coleman S. J. Huang Y. Savich G. L.. (2010). MapSplice: accurate mapping of RNA-seq reads for splice junction discovery. Nucleic Acids Res. 38:e178. 10.1093/nar/gkq622 20802226 PMC2952873

[B94] Wang L. Dowell R. D. Yi R. (2013). Genome-wide maps of polyadenylation reveal dynamic mRNA 3'-end formation in mammalian cell lineages. RNA 19, 413–425. 10.1261/rna.035360.112 23325109 PMC3677251

[B95] Wang L. Hu X. Wang P. Shao Z. M. (2016). The 3'UTR signature defines a highly metastatic subgroup of triple-negative breast cancer. Oncotarget 7, 59834–59844. 10.18632/oncotarget.10975 27494850 PMC5312352

[B96] Wang W. Wei Z. Li H. (2014). A change-point model for identifying 3'UTR switching by next-generation RNA sequencing. Bioinformatics 30, 2162–2170. 10.1093/bioinformatics/btu189 24728858 PMC4103598

[B97] Willis D. E. Xu M. Donnelly C. J. Tep C. Kendall M. Erenstheyn M.. (2011). Axonal localization of transgene mRNA in mature PNS and CNS neurons. J. Neurosci. 31, 14481–14487. 10.1523/JNEUROSCI.2950-11.2011 21994364 PMC3205917

[B98] Wu J. Akerman M. Sun S. Mccombie W. R. Krainer A. R. Zhang M. Q. (2011). SpliceTrap: a method to quantify alternative splicing under single cellular conditions. Bioinformatics 27, 3010–3016. 10.1093/bioinformatics/btr508 21896509 PMC3198574

[B99] Xia Z. Donehower L. A. Cooper T. A. Neilson J. R. Wheeler D. A. Wagner E. J.. (2014). Dynamic analyses of alternative polyadenylation from RNA-seq reveal a 3'-UTR landscape across seven tumour types. Nat. Commun. 5:5274. 10.1038/ncomms6274 25409906 PMC4467577

[B100] Yasuda M. Tanaka Y. Ryu M. Tsuda S. Nakazawa T. (2014). RNA sequence reveals mouse retinal transcriptome changes early after axonal injury. PLoS ONE 9:e93258. 10.1371/journal.pone.0093258 24676137 PMC3968129

[B101] Yates A. Akanni W. Amode M. R. Barrell D. Billis K. Carvalho-Silva D.. (2016). Ensembl 2016. Nucleic Acids Res. 44, D710–D716. 10.1093/nar/gkv1157 26687719 PMC4702834

[B102] Ye C. Long Y. Ji G. Li Q. Q. Wu X. (2018). APAtrap: identification and quantification of alternative polyadenylation sites from RNA-seq data. Bioinformatics 34, 1841–1849. 10.1093/bioinformatics/bty029 29360928

[B103] Yeh H. S. Zhang W. Yong J. (2017). Analyses of alternative polyadenylation: from old school biochemistry to high-throughput technologies. BMB Rep. 50, 201–207. 10.5483/BMBRep.2017.50.4.019 28148393 PMC5437964

[B104] Zhang H. (2016). Overview of sequence data formats. Methods Mol. Biol. 1418, 3–17. 10.1007/978-1-4939-3578-9_1 27008007

[B105] Zhang J. Wei Z. (2016). An empirical Bayes change-point model for identifying 3' and 5' alternative splicing by next-generation RNA sequencing. Bioinformatics 32, 1823–1831. 10.1093/bioinformatics/btw060 26873932

[B106] Zhang L. Li X. Zhou R. Xing G. (2006). Possible role of potassium channel, big K in etiology of schizophrenia. Med. Hypotheses 67, 41–43. 10.1016/j.mehy.2005.09.055 16446048

