# Supplementary material for: Detection of Differentially Expressed Cleavage Site Intervals Within 3′ Untranslated Regions Using CSI-UTR Reveals Regulated Interaction Motifs
Source: Front Genet. 2019 Mar 12;10:182. doi: 10.3389/fgene.2019.00182 (PMC6422928; doi:10.3389/fgene.2019.00182)
Supplement: Supplementary file 1 [file Data_Sheet_1.pdf]

Supplementary Material

Detection of Differentially Expressed Cleavage Site Intervals within 3' Untranslated Regions using CSI-UTR Reveals Regulated Interaction Motifs

Benjamin J. Harrison[1,2,3], Juw Won Park[3,4], Cynthia Gomes[2,5], Jeffrey C. Petruska[2,5,6], Matthew R. Sapio[7], Michael J. Iadarola[7], Julia H. Chariker[2,3], and Eric C. Rouchka[3,4,*]

*** Correspondence:**
Dr. Eric C. Rouchka
eric.rouchka@louisville.edu

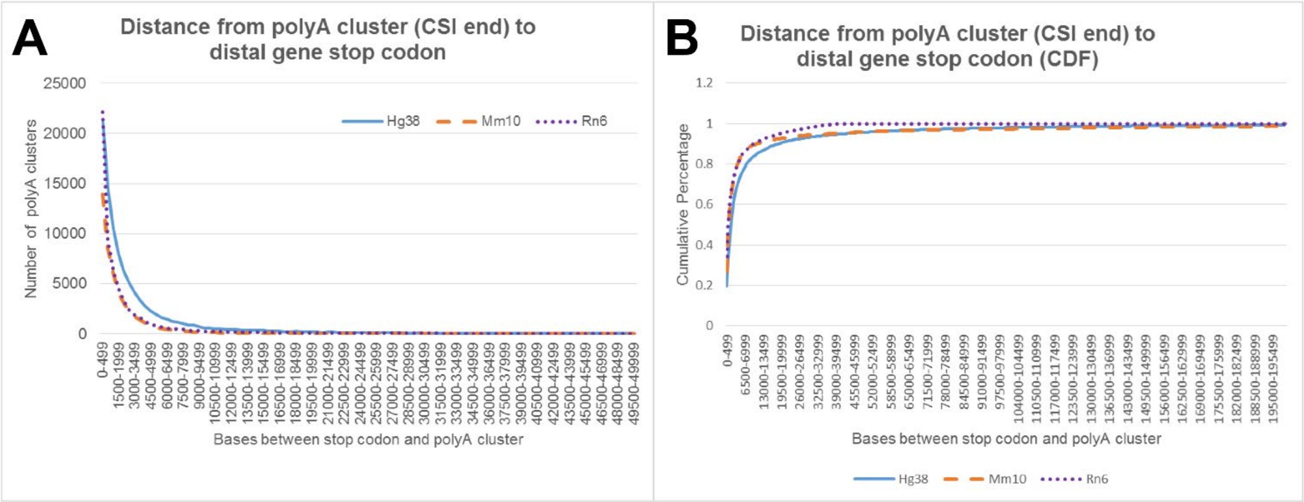

Supplemental Figure 1: Distance from distal-most stop codon to detected polyA cluster. (A) shows the number of polyA clusters occurring within a 500 bp bin while **(B)** shows the cumulative distribution. Over 90% of all identified polyA clusters are located within 20kb of the distal-most stop codon for all three species, with approximately 5% of human and mouse polyA clusters extending past 40kb.

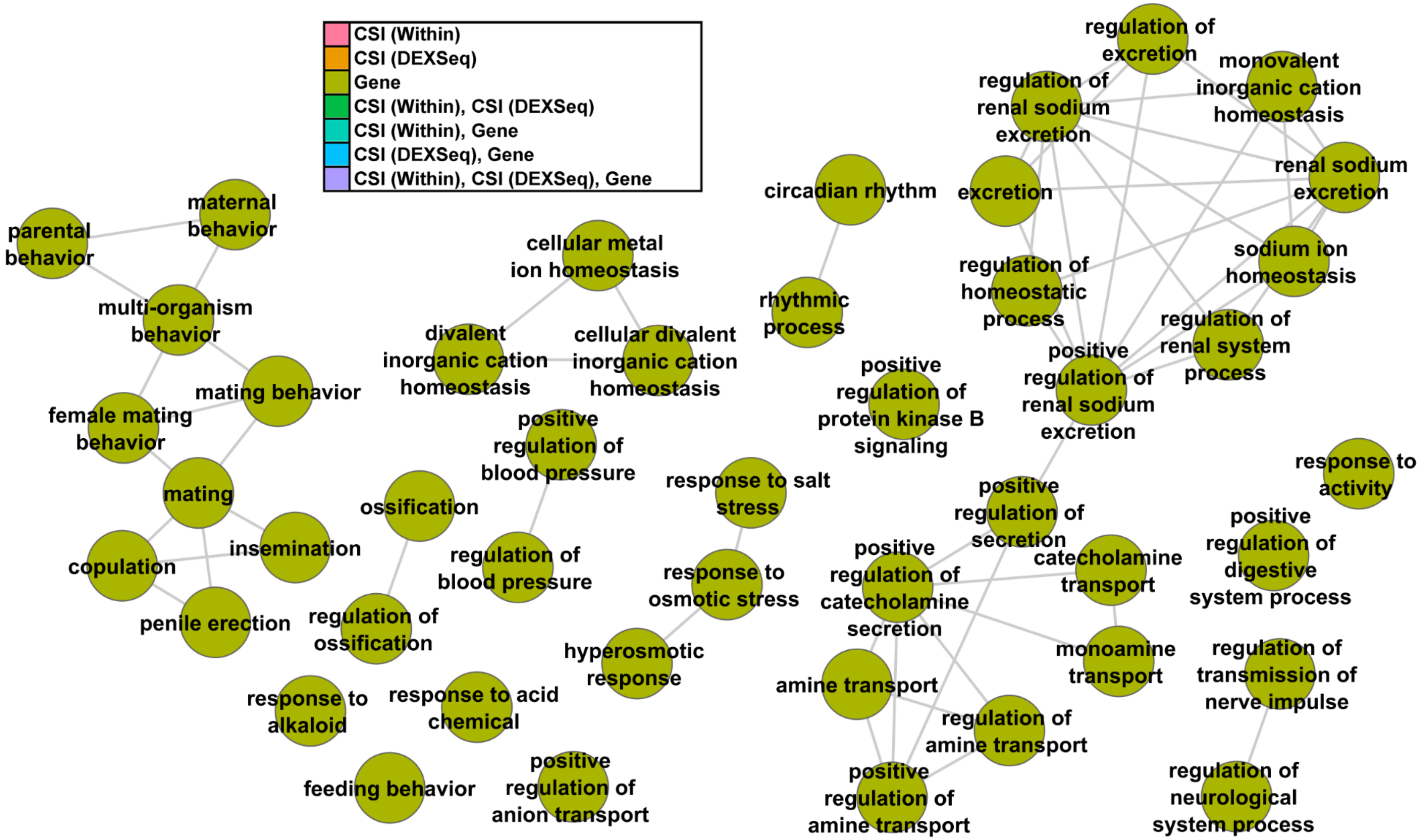

Supplemental Figure 2: Gene Ontology Biological Process enrichment analysis using categoryCompare for human (Hs) comparison, part 1. Categories shown pass an FDR < 0.05. Nodes represent individual GO:BP categories while edges represent connections between GO:BP categories with shared significantly differentially expressed gene lists of 90% or greater.

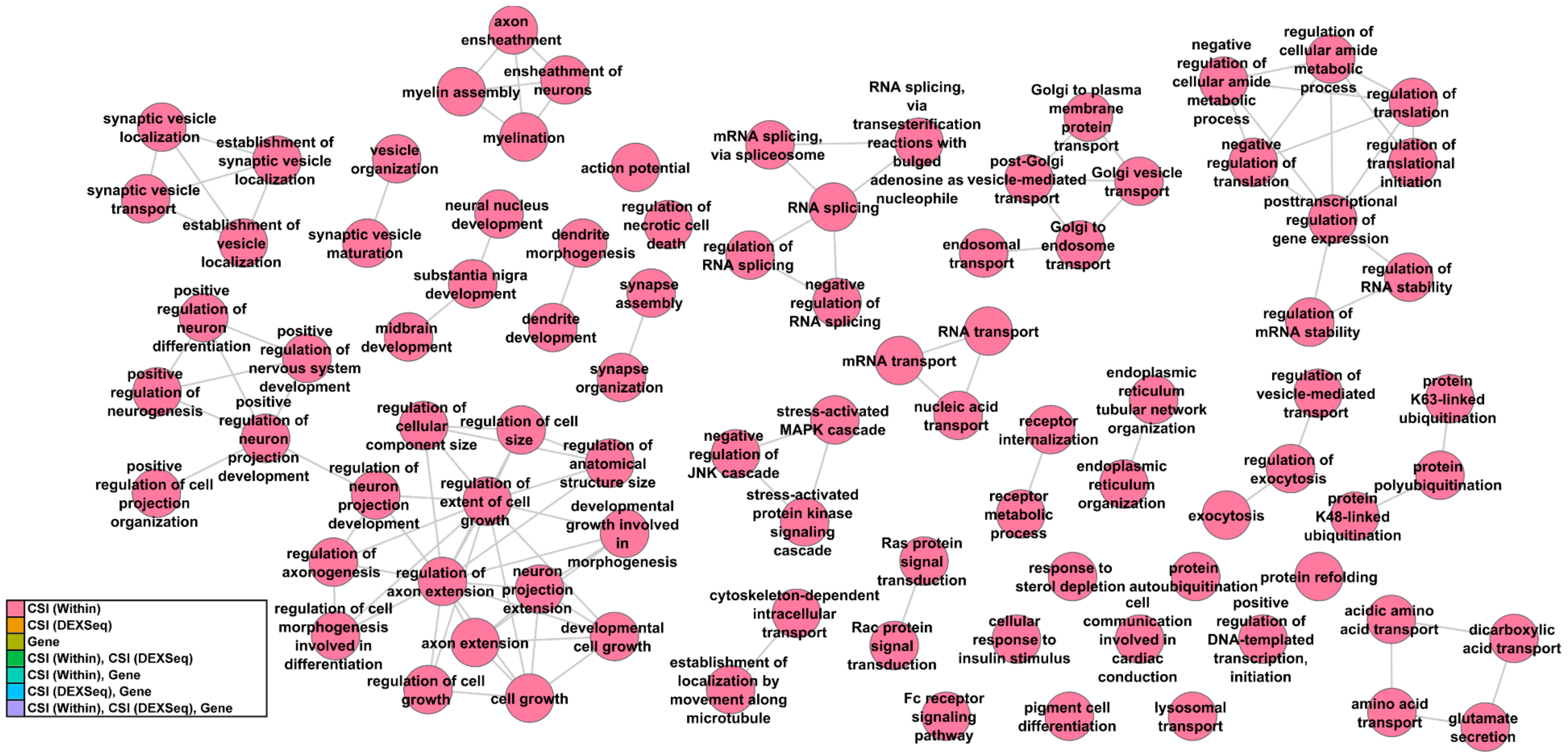

Supplemental Figure 3: Gene Ontology Biological Process enrichment analysis using categoryCompare for human (Hs) comparison, part 2. Categories shown pass an FDR < 0.05. Nodes represent individual GO:BP categories while edges represent connections between GO:BP categories with shared significantly differentially expressed gene lists of 90% or greater.

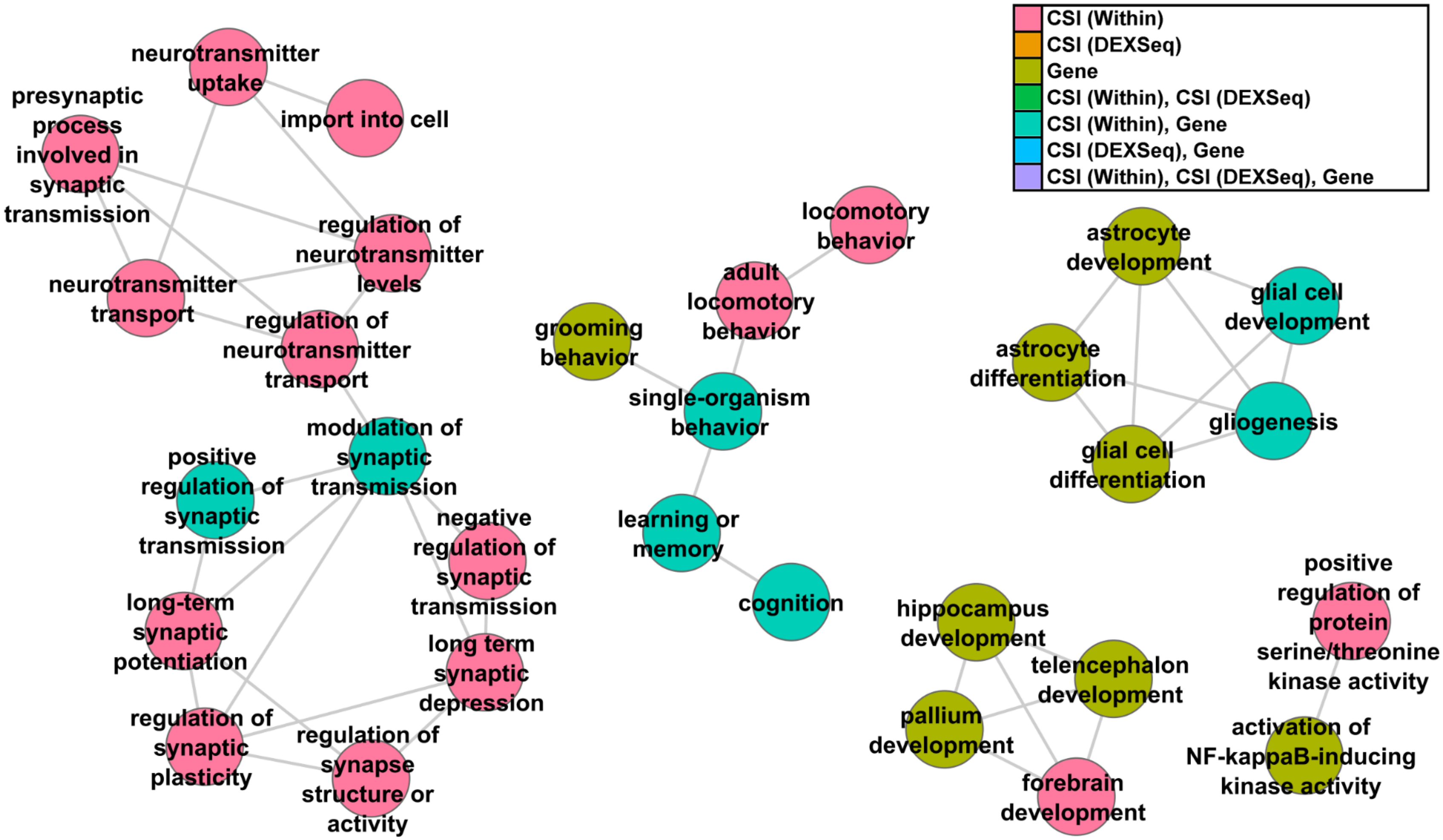

Supplemental Figure 4: Gene Ontology Biological Process enrichment analysis using categoryCompare for human (Hs) comparison, part 3. Categories shown pass an FDR < 0.05. Nodes represent individual GO:BP categories while edges represent connections between GO:BP categories with shared significantly differentially expressed gene lists of 90% or greater.

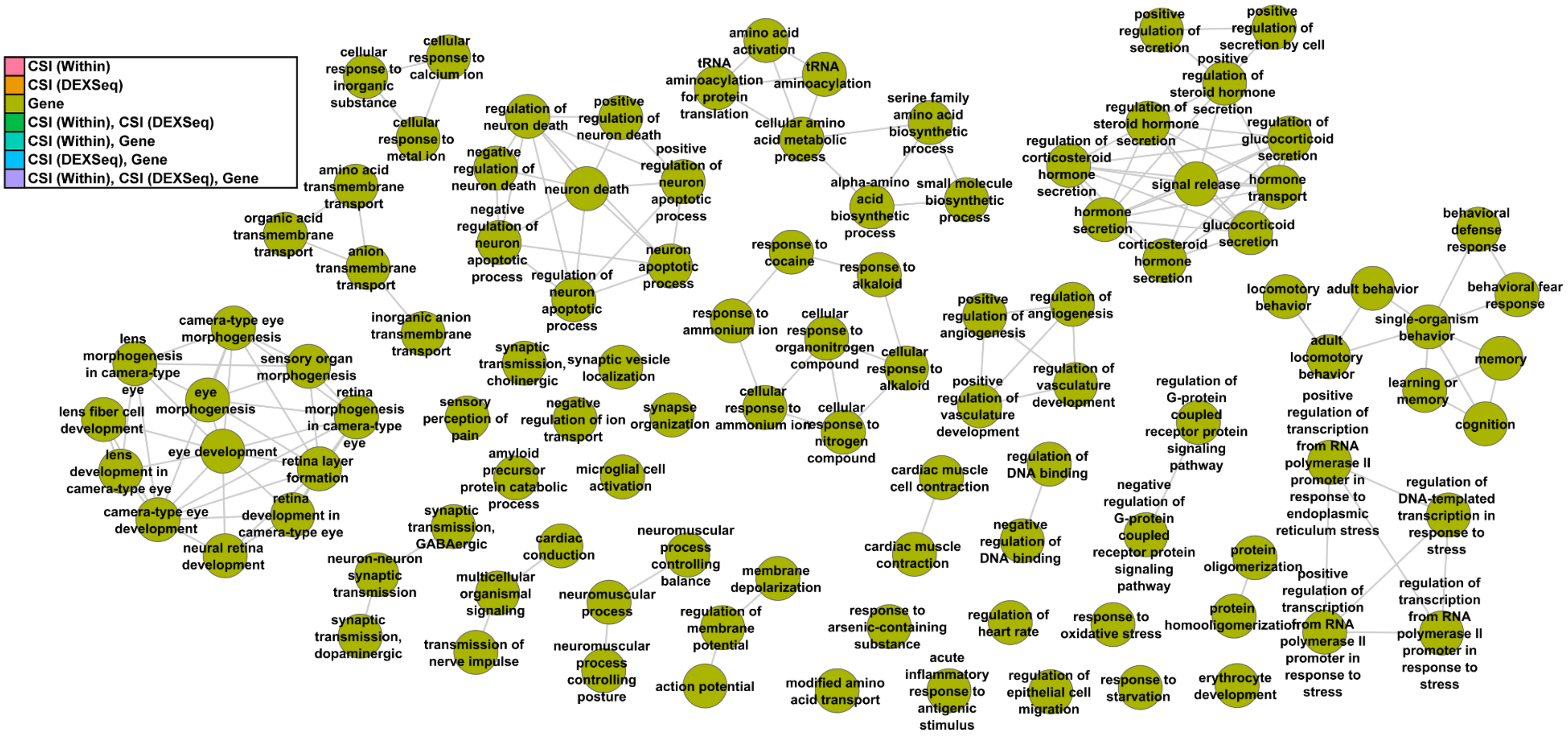

Supplemental Figure 5: Gene Ontology Biological Process enrichment analysis using categoryCompare for mouse (Mm) comparison, part 1. Categories shown pass an FDR < 0.05. Nodes represent individual GO:BP categories while edges represent connections between GO:BP categories with shared significantly differentially expressed gene lists of 90% or greater.

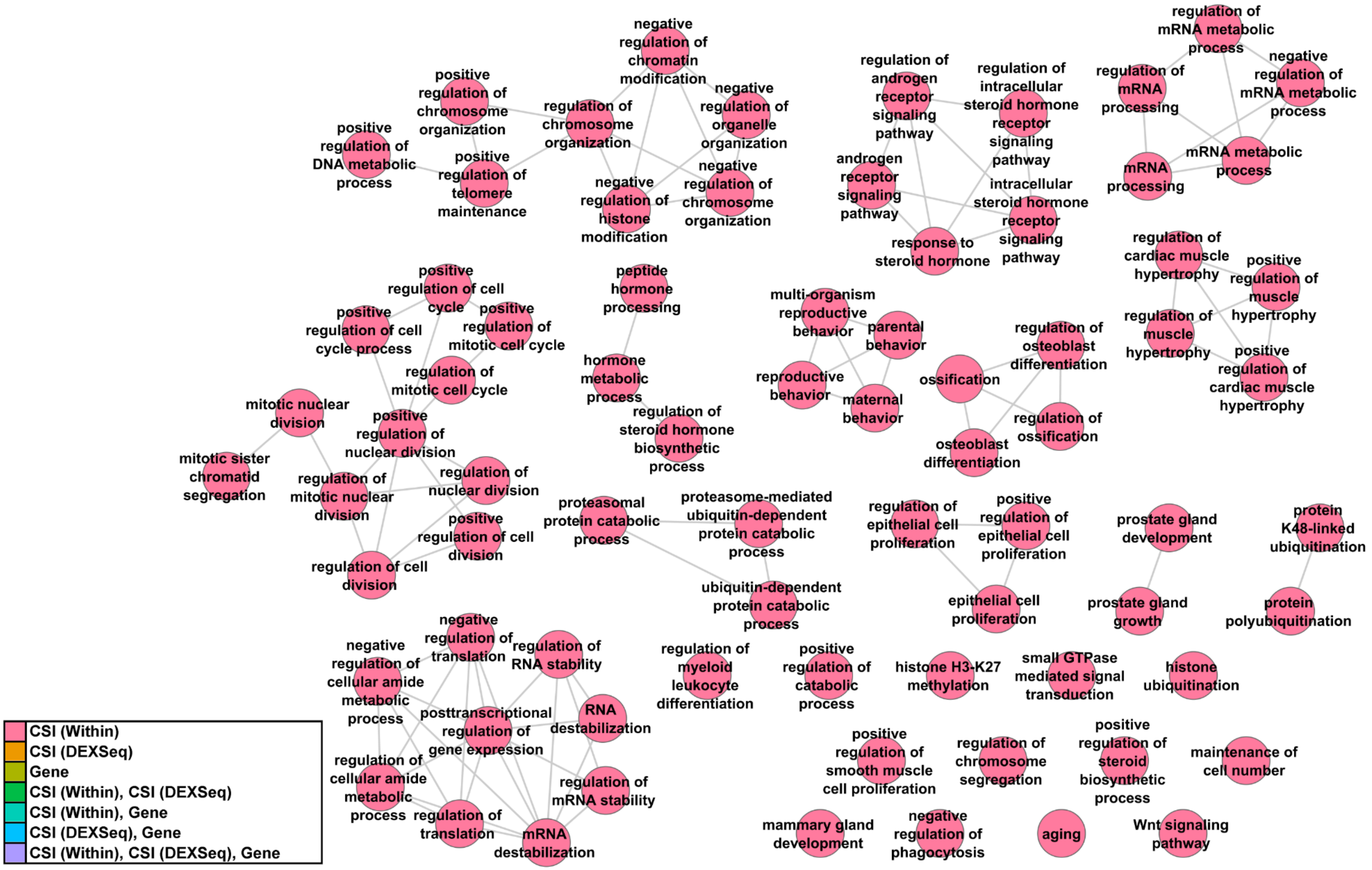

Supplemental Figure 6: Gene Ontology Biological Process enrichment analysis using categoryCompare for mouse (Mm) comparison, part 2. Categories shown pass an FDR < 0.05. Nodes represent individual GO:BP categories while edges represent connections between GO:BP categories with shared significantly differentially expressed gene lists of 90% or greater.

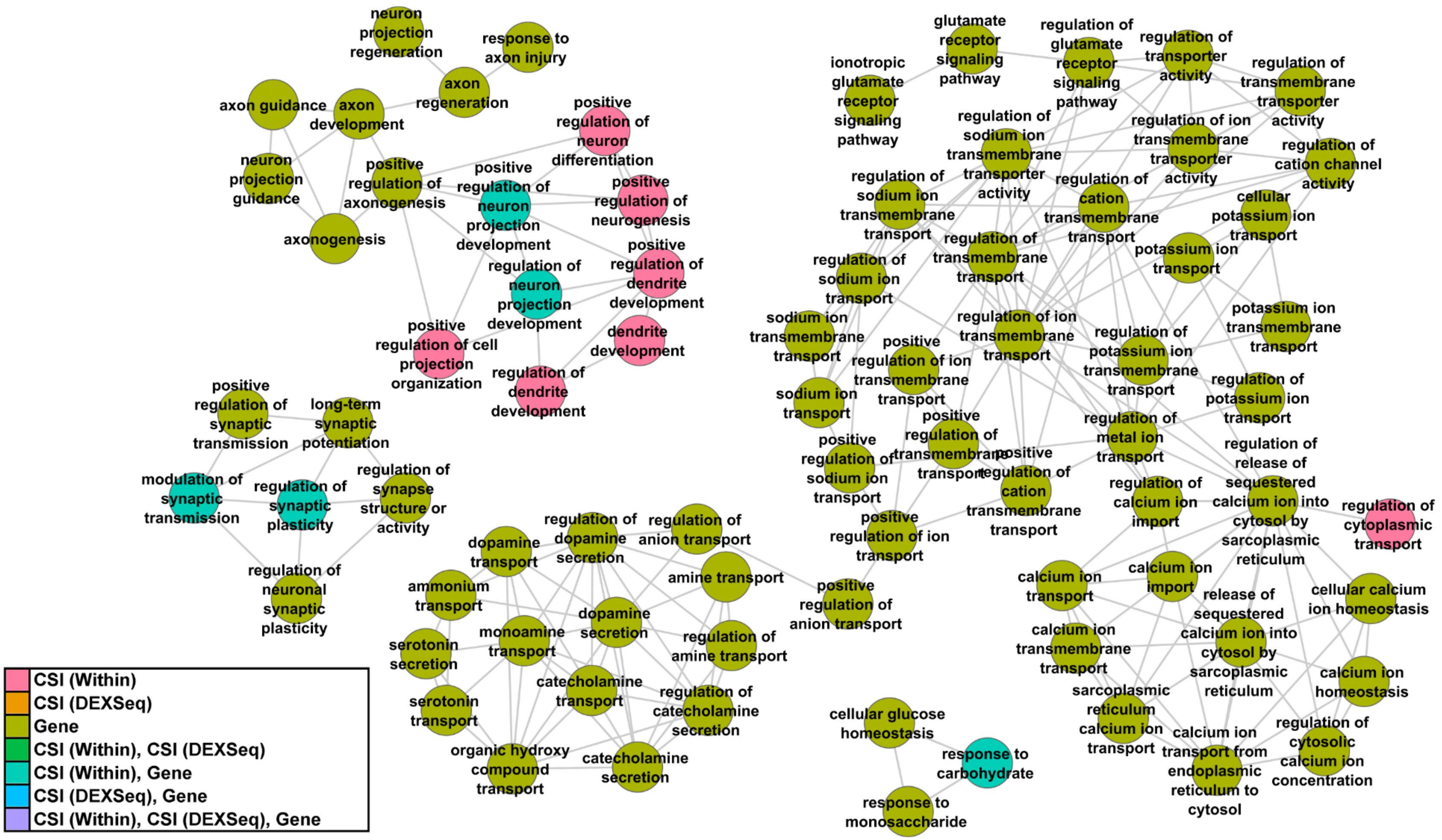

Supplemental Figure 7: Gene Ontology Biological Process enrichment analysis using categoryCompare for mouse (Mm) comparison, part 3. Categories shown pass an FDR < 0.05. Nodes represent individual GO:BP categories while edges represent connections between GO:BP categories with shared significantly differentially expressed gene lists of 90% or greater.

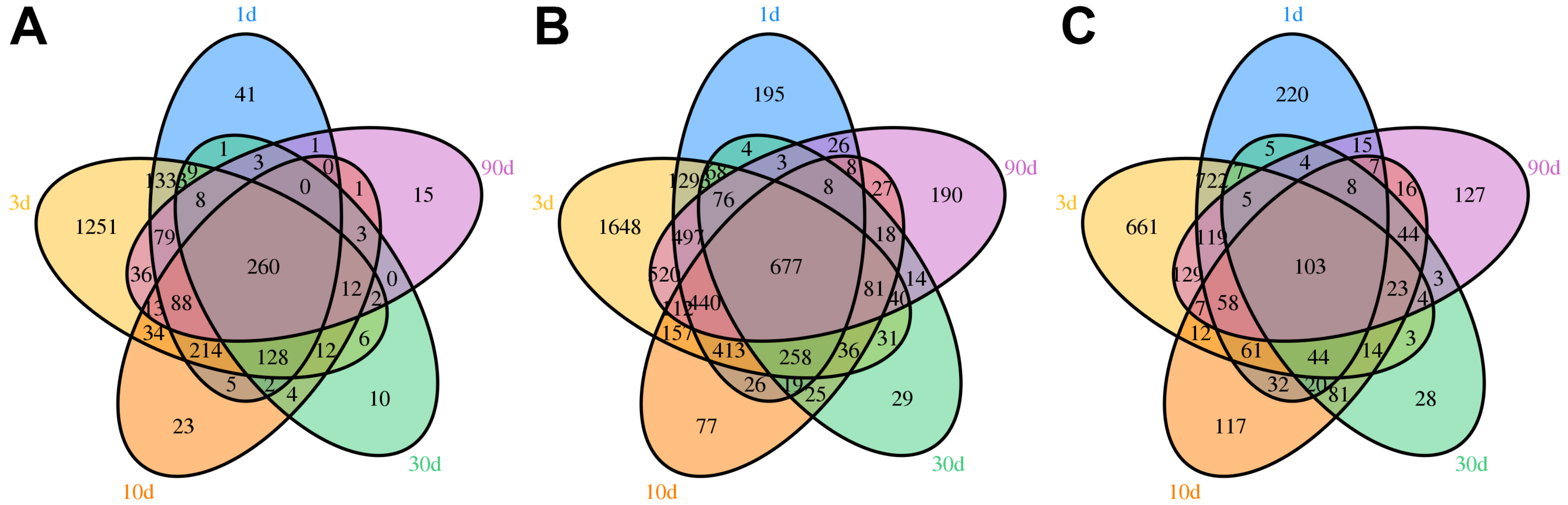

Supplemental Figure 8: Venn diagrams for shared differentially expressed genes/genes with CSIs using (A) DE-CSIs using WITHIN; (B) DE-CSIs using DEXSeq; (C) DEGs.

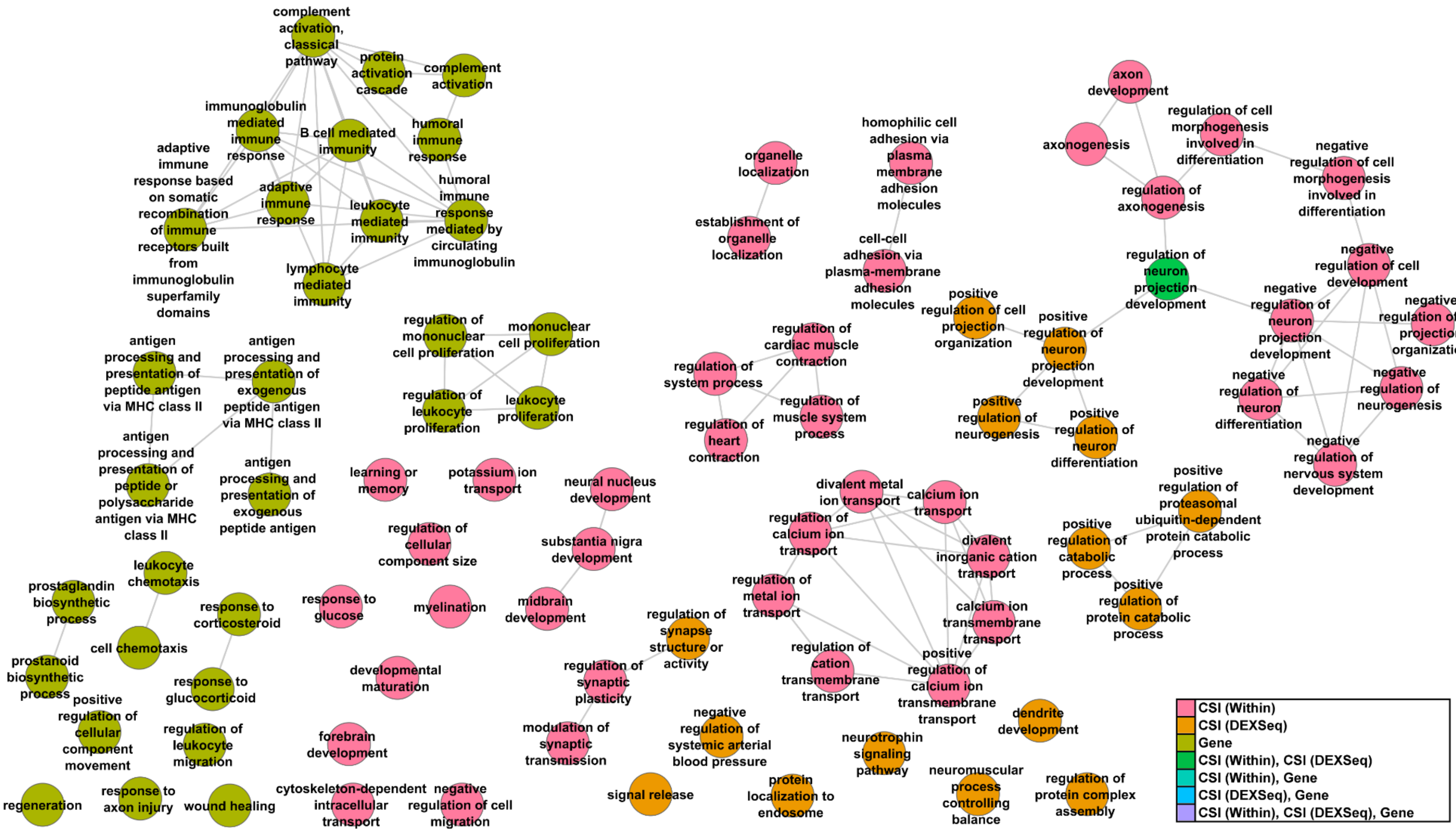

Supplemental Figure 9: Gene Ontology Biological Process enrichment analysis using categoryCompare for common differentially expressed components for rat (Rn) comparisons. Categories shown pass an FDR < 0.05. Nodes represent individual GO:BP categories while edges represent connections between GO:BP categories with shared significantly differentially expressed gene lists of 90% or greater.

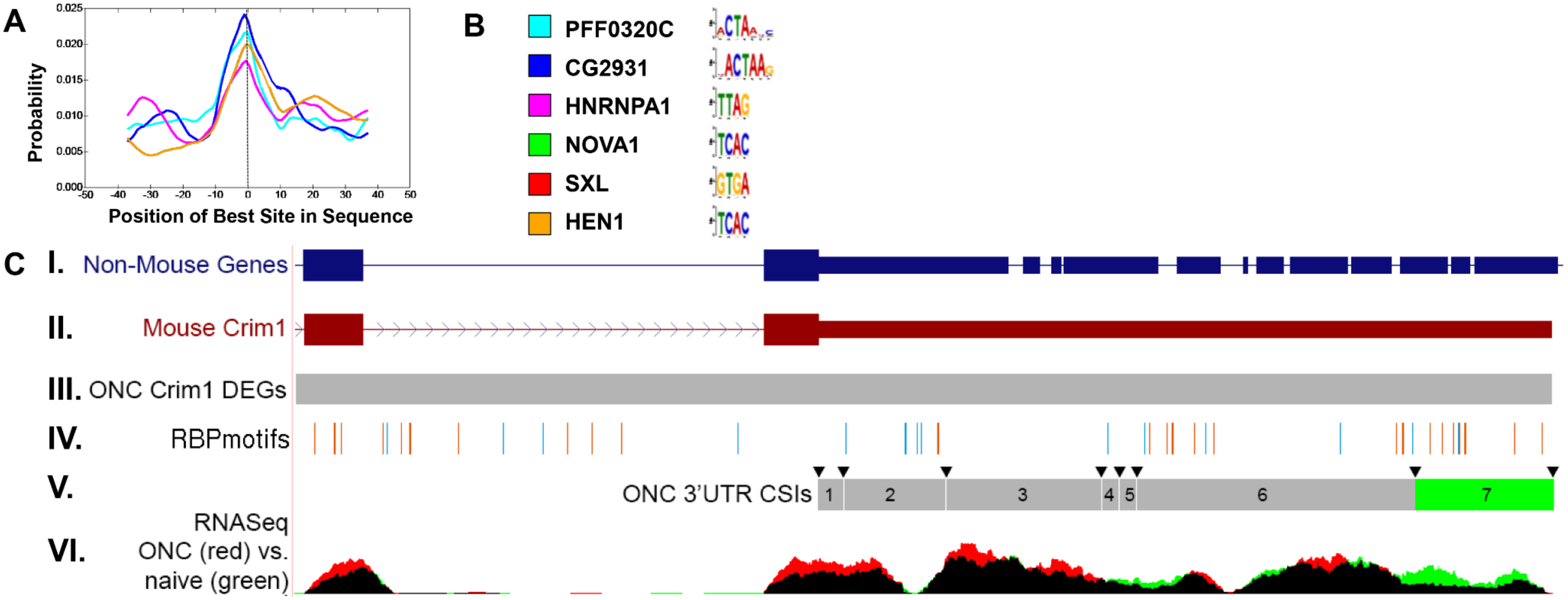

Supplemental Figure 10: Enriched motifs for mouse optic nerve crush (ONC) differentially expressed CSIs. Shown are enriched RNA binding protein (RBP) motifs (A) overlapping the boundaries of significantly changed CSI sites with the overlapping motifs reported in (B). Panel (C) shows an example of the CSI-UTR analysis within the mouse *Crim1* region encompassing the last two coding exons and the 3'UTR (see Figure 6 for the corresponding rat region). Subpanels I and II show that the mouse *Crim1* 3'UTR annotation extends in agreement with non-mouse *Crim1* homologs. *Crim1* is not differentially expressed at the gene level, as represented by the gray bar in subpanel III. The location of the RBP motifs from (A) and (B) are shown in subpanel IV, which are in general clustered around the CSI boundaries shown by the triangles in subpanel V. The resulting RNASeq data from the ONC model indicates a higher (but non-significant) expression in ONC (red) vs. naïve (green). However, the ONC transcripts are generally shorter, ending after CSI 6 as illustrated in subpanels V and VI. This is in contrast to the naïve transcripts that extend to the end of CSI 7 as indicated by the green portion in subpanels V and VI.

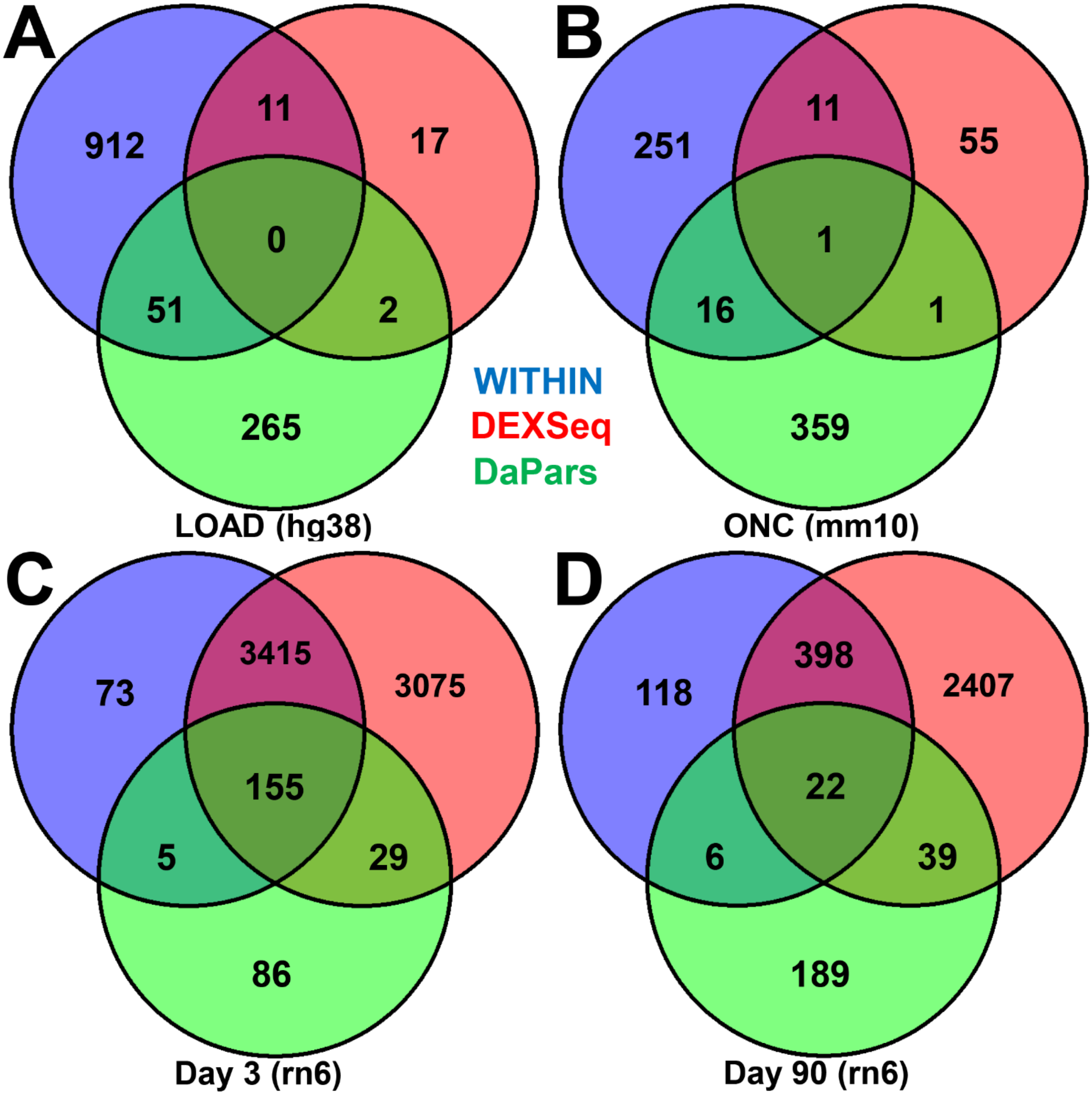

Supplemental Figure 11: Comparison of Ensembl genes with detected CSI/APA events within the CSI-UTR WITHIN method (blue), CSI-UTR DEXSeq method (red) and DaPars (green). Shown are the results for **(A)** the human LOAD dataset, **(B)** mouse ONC dataset, **(C)** rat dataset at day (3) and **(D)** at day 90. Numbers represent the number of unique Ensembl gene identifiers found in each case. Each gene may have multiple events within them.

Supplemental Table 1: Top genes according to maximum number of cleavage site intervals (CSIs).

Gene	Max CSIs	Hs CSIs	Mm CSIs	Rn CSIs
KCTD12	77	77	11	--
PTH2R	69	69	1	2
PCDH10	51	51	3	17
GNL3L	50	50	2	5
MAF	50	4	16	50
FLRT2	49	49	11	6
Frmd8	49	8	1	49
SLITRK1	49	49	3	22
SPCS3	48	48	7	7
GDAP2	47	47	12	11
PCDH17	47	21	19	47
ZNF514	47	47	--	--
ZWINT	47	47	7	16
MTERF1	46	46	--	4
TMEM161B	46	46	4	25
PDZRN4	45	9	6	45
SMAD2	44	44	13	13
UBE2V2	43	43	10	13
ZMAT3	43	43	7	9
DR1	42	42	5	--
AABR07050561.1	41	--	--	41
ARL5A	41	41	12	10
CRIPT	40	40	6	12
PCDH9	40	11	6	40
RAB18	40	40	14	11
QKI	39	39	18	26
G6PC	38	38	3	1
ACTR3C	37	37	--	--
FAM46A	37	37	3	9
NCAM2	37	11	12	37
PPP1R3A	37	37	13	2
SNX18	37	37	10	8
SOD2	37	37	8	17

Supplemental Table 2: Enriched GO:BP categories for DEGs from human (Hs) comparison (FDR ≤ 0.001).

GO ID	Description	Count	P-value	FDR
GO:0044708	single-organism behavior	11	7.61E-08	0

Supplemental Table 3: Enriched GO:BP categories for DE-CSIs using the WITHIN pipeline for the human (Hs) comparison (FDR ≤ 0.001).

GO ID	Description	Count	P-value	FDR
GO:0021762	substantia nigra development	12	1.53E-06	0.001
GO:0048667	cell morphogenesis involved in neuron differentiation	53	1.58E-06	0.000952

Supplemental Table 4: Enriched GO:BP categories for DEGs for the mouse (Mm) comparison (FDR ≤ 0.001).

GO ID	Description	Count	P-value	FDR
GO:0034765	regulation of ion transmembrane transport	28	1.74E-12	0
GO:0034762	regulation of transmembrane transport	28	4.30E-12	0
GO:0098655	cation transmembrane transport	30	8.79E-11	0
GO:0098662	inorganic cation transmembrane transport	28	5.44E-10	0
GO:0015672	monovalent inorganic cation transport	25	4.61E-09	0
GO:0044708	single-organism behavior	25	2.49E-08	0
GO:1904062	regulation of cation transmembrane transport	17	3.10E-08	0
GO:0032409	regulation of transporter activity	16	4.47E-08	0
GO:0010959	regulation of metal ion transport	21	5.01E-08	0
GO:0032412	regulation of ion transmembrane transporter activity	15	7.24E-08	0
GO:0050804	modulation of synaptic transmission	20	7.47E-08	0
GO:0022898	regulation of transmembrane transporter activity	15	1.12E-07	0
GO:0035637	multicellular organismal signaling	12	2.07E-07	0
GO:0015844	monoamine transport	10	3.52E-07	0
GO:0001508	action potential	12	3.90E-07	0
GO:0007270	neuron-neuron synaptic transmission	13	5.28E-07	0
GO:0043010	camera-type eye development	18	7.09E-07	0
GO:0042391	regulation of membrane potential	20	7.38E-07	0
GO:0001654	eye development	19	1.37E-06	0
GO:0050905	neuromuscular process	11	1.74E-06	0
GO:0043618	regulation of transcription from RNA polymerase II promoter in response to stress	7	2.26E-06	0
GO:0043620	regulation of DNA-templated transcription in response to stress	7	4.33E-06	0
GO:0035725	sodium ion transmembrane transport	9	7.36E-06	0
GO:0050806	positive regulation of synaptic transmission	11	8.49E-06	0
GO:0006837	serotonin transport	5	9.30E-06	0.000328
GO:0006813	potassium ion transport	13	1.16E-05	0.000645
GO:0048593	camera-type eye morphogenesis	10	1.30E-05	0.000635
GO:0071805	potassium ion transmembrane transport	11	1.53E-05	0.000909
GO:0071804	cellular potassium ion transport	11	1.63E-05	0.000896
GO:0015872	dopamine transport	6	1.67E-05	0.00087

Supplemental Table 5: Enriched GO:BP categories for DE-CSIs using the WITHIN pipeline for the mouse (Mm) comparison ($p \leq 0.05$).

GO ID	Description	Count	P-value	FDR
GO:0034248	regulation of cellular amide metabolic process	15	6.11E-06	0
GO:0006417	regulation of translation	14	9.82E-06	0.000417
GO:0050804	modulation of synaptic transmission	10	0.005317	0.062026

Supplemental Table 6: Enriched GO:BP categories for DE-CSIs using the DEXSeq pipeline for the mouse (Mm) comparison ($p \leq 0.05$).

GO ID	Description	Count	P-value	FDR
GO:0050905	neuromuscular process	4	0.000624	0.187273
GO:0098662	inorganic cation transmembrane transport	7	0.000833	0.21
GO:0098655	cation transmembrane transport	7	0.001141	0.211111
GO:0071805	potassium ion transmembrane transport	4	0.001441	0.22381
GO:0071804	cellular potassium ion transport	4	0.001477	0.204348
GO:0007270	neuron-neuron synaptic transmission	4	0.001589	0.2096
GO:0006837	serotonin transport	2	0.001927	0.189697
GO:0006813	potassium ion transport	4	0.004326	0.302319
GO:0015872	dopamine transport	2	0.006517	0.284719
GO:0042391	regulation of membrane potential	5	0.006979	0.338505
GO:0015672	monovalent inorganic cation transport	5	0.011826	0.346667
GO:0050804	modulation of synaptic transmission	4	0.0203	0.39747
GO:0015844	monoamine transport	2	0.028922	0.41854
GO:0034765	regulation of ion transmembrane transport	4	0.032102	0.431361
GO:0034762	regulation of transmembrane transport	4	0.036082	0.440574
